# Targeting TREM2^+^ and TIMD4^+^ Macrophages Reprograms the Tumor Microenvironment and Enhances PD-1 Blockade Efficacy in Intrahepatic Cholangiocarcinoma

**DOI:** 10.34133/cancomm.0056

**Published:** 2026-10-09

**Authors:** Yao Li, Hongwen Liu, Lei Xu, Lili Huang, Hui Xue, Tao Lu, Yuxuan Lin, Liangrui Chen, Jianwei Liu, Shilei Bai, Yong Xia, Lei Wang, Hui Dong, Feng Shen, Kui Wang

**Affiliations:** ^1^ Department of Hepatic Surgery II, Eastern Hepatobiliary Surgery Hospital, Naval Medical University, Shanghai, P. R. China.; ^2^ Department of Gastroenterology, Nanjing Drum Tower Hospital, The Affiliated Hospital of Nanjing University Medical School, Nanjing, Jiangsu, P. R. China.; ^3^ Department of Hepatic Surgery IV, Eastern Hepatobiliary Surgery Hospital, Naval Medical University, Shanghai, P. R. China.; ^4^ Department of Pathology, Eastern Hepatobiliary Surgery Hospital, Naval Medical University, Shanghai, P. R. China.

## Abstract

**Background:** Intrahepatic cholangiocarcinoma (ICC) is the second most common primary liver cancer. Macrophages are crucial components of the tumor microenvironment (TME). Triggering receptor expressed on myeloid cells 2-positive (TREM2^+^) macrophages have recently emerged as potent pro-tumor progress players in several cancers. However, the heterogeneity and tumor-promoting mechanisms of macrophages especially TREM2^+^ macrophages in ICC remain systematically unexplored. Therefore, this study aimed to systematically elucidate the heterogeneity of macrophages in ICC, with a specific focus on the pro-tumor role of TREM2^+^ macrophages. **Methods:** We collected tumor tissues, adjacent nontumor tissues, and tumor-adjacent border tissues from 16 ICC patients. These samples were subjected to single-cell RNA sequencing and spatial transcriptomic sequencing, followed by comprehensive analysis. The findings were further validated through flow cytometry, immunohistochemistry, multiplex immunofluorescence, and in vitro cellular assays. Furthermore, we established an ICC model using genetically engineered (*Trem2^−/−^*) mice as a validation cohort to corroborate the discoveries from human samples. **Results:** Macrophages are important prognostic factors in ICC, with functional heterogeneity. TREM2^+^ macrophages in tumor tissues promote immune suppression by exhausting CD8^+^ T cells and inducing the generation of regulatory T cells to produce. At the tumor border region, TREM2^+^ macrophages enhance ICC invasion via the urokinase-type plasminogen activator–urokinase-type plasminogen activator receptor axis and induce hepatic stellate cell conversion to myofibroblastic cancer-associated fibroblast, forming a physical immune barrier that impedes CD8^+^ T cell infiltration in tumor tissues. TIMD4^+^ macrophages in adjacent tissues also suppress CD8^+^ T cell function. Combining anti-Trem2 and anti-Timd4 with anti-Pd-1 antibodies significantly improved treatment outcomes in the ICC mice. **Conclusions:** TREM2^+^ and TIMD4^+^ macrophages are key regulators of the immunosuppressive TME in ICC. Targeting Trem2 and Timd4 enhances the effectiveness of anti-Pd-1 therapy in ICC mouse models.

## Background

Intrahepatic cholangiocarcinoma (ICC) accounts for 10% to 15% of all primary liver cancers [1,2]. Patients with ICC face a dire prognosis (5-year overall survival <10%), attributable to cancer’s aggressive biology, challenges in early detection, and limited efficacy of standard chemotherapies [3]. Although immune checkpoint blockade therapies targeting programmed cell death protein 1/programmed death-ligand 1 (PD-1/PD-L1) have achieved breakthroughs in various solid tumors, their response rates in ICC remain suboptimal [4], suggesting potential undiscovered immunosuppressive mechanisms within the tumor immune microenvironment (TIME).

As critical regulators of TIME, tumor-associated macrophages (TAMs) have become a hot topic of research because of their functional heterogeneity and tumor-promoting potential [5]. Nevertheless, the characteristics of distinct TAM subtypes within tumors and their roles in immune evasion [6–9], tumor invasion [10], and recruiting regulatory T cells (Tregs; [11,12]) remain incompletely understood. For instance, TAMs facilitate tumor metastasis via ferroptosis resistance in hepatocellular carcinoma [13]. Triggering receptor expressed on myeloid cells 2 (TREM2) has been shown to promote immune escape by suppressing CD8^+^ T cell function in breast cancer [14] and hepatocellular carcinoma [15]. Furthermore, TREM2^+^ macrophage drives natural killer (NK) cell dysfunction in lung cancer [16]. In the nonneoplastic disease, TREM2^+^ macrophage was associated with liver cirrhosis [17]. These suggest that TREM2^+^ macrophages regulate immune effector processes by modulating T cell function and may also influence the tumor microenvironment (TME) through orchestrating matrix remodeling. However, systematic investigations into the spatial distribution patterns of TREM2^+^ macrophages and their interaction networks with stromal cells in ICC remain unclear. In addition, most studies are limited to the inside of the tumor, and the potential involvement of peritumoral region in establishing immunosuppressive microenvironments remains undefined.

The current clinical treatment of ICC faces several challenges: targeted therapies (isocitrate dehydrogenase 1 [IDH1]/fibroblast growth factor receptor 2 inhibitors) are only effective in limited genetic subtypes. The TOPAZ-1 clinical trial (NCT03875235) demonstrated that PD-L1 inhibitors combined with chemotherapy provided greater overall survival benefits compared to chemotherapy alone [18]. However, the median overall survival (mOS) was below 15% (median follow-up period, 13.7 months). In addition, although the KEYNOTE966 (NCT04003636) clinical trial showed that the mOS of the pembrolizumab-chemotherapy group was all improved relative to the placebo-chemotherapy group, the mOS could only reach 12.7 months [19]. Recent clinical trials (NCT03951597) evaluating PD-1 inhibitors combined with chemotherapy (gemcitabine combined with oxaliplatin) and targeted therapies (lenvatinib) reported that the 2-year overall survival rate was 49.8% [20]. However, this is a single-arm study lacking a more reliable level of evidence, although the combination of immunotherapy and chemotherapy has emerged as a first-line treatment for ICC. However, the rapid development of drug resistance and severe adverse effects associated with this approach have further limited its efficacy. Consequently, there is an urgent need to investigate novel therapeutic strategies for ICC, particularly focusing on enhancing the effectiveness of immunotherapy.

The primary objective of this study is to elucidate the spatial and functional heterogeneity of macrophages within the TME of ICC, with a specific focus on the mechanisms by which TREM2^+^ macrophages exert tumor-promoting roles. Additionally, this research aimed to explore macrophage-targeted therapeutic strategies for ICC.

## Materials and Methods

### Human subjects

Human ICC samples were obtained from surgically resected specimens rather than tissue biopsies. In total, tissue specimens from 70 unique patients with pathologically confirmed ICC were collected at The Third Affiliated Hospital of Naval Medical University. Written informed consent was obtained from all patients before sample collection, and the study was approved by the Institutional Ethics Committee of The Third Affiliated Hospital of Naval Medical University (registration number: EHBHKY2020-K-022). None of the patients had received preoperative anticancer treatment, and all cases were pathologically confirmed as ICC.

Fresh surgical specimens were used for single-cell RNA sequencing (scRNA-seq), flow cytometry analysis, and macrophage isolation. Specifically, 16 in-house ICC cases were used for scRNA-seq, and an additional 8 ICC cases from the public dataset GSE138709 were included, resulting in a total of 24 cases for scRNA-seq analysis. Five cases were used for flow cytometry analysis, and 14 cases were used for macrophage isolation. Formalin-fixed, paraffin-embedded tissue sections or archival pathological sections were used for immunohistochemistry (IHC), immunofluorescence, and multiplex immunofluorescence (mIF) analyses, including 39 cases for IHC and 23 cases for mIF. Detailed clinicopathological characteristics and the corresponding experimental applications of each case are provided in Table S1.

The inclusion criteria were as follows: patients with pathologically confirmed ICC; patients who underwent surgical resection; availability of adequate tumor tissue and, when required, matched adjacent normal or border tissues; no preoperative anticancer treatment; and written informed consent. The exclusion criteria included patients who received chemotherapy, radiotherapy, targeted therapy, immunotherapy, or other anticancer treatments before surgery; patients with mixed hepatocellular-cholangiocarcinoma or other malignancies; insufficient or poor-quality tissue samples; and incomplete clinical information.

For regional sampling, tumor tissues were collected from areas more than 0.5 cm inside the tumor boundary; border tissues included the interface between the tumor and adjacent nontumor tissue, with approximately equal proportions of both regions; and adjacent normal tissues were collected more than 1 cm away from the tumor boundary and avoided areas affected by ultrasonic scalpel-induced thermal damage.

### Cells and cell culture

The human cholangiocarcinoma cell lines CCLP1 (catalog no. QuiCell-C1964, QuiCellBio), HuCCT1 (catalog no. ZQ0477, ZQXzbio), RBE (catalog no. ZQ0247, ZQXzbio), and HCCC-9810 (catalog no. ZQ0250, ZQXzbio), as well as the human intrahepatic biliary epithelial cell line HiBEpiC (catalog no. ZQY009, ZQXzbio) and fibroblast cell line LX-2 (catalog no. ZQ0026, ZQXzbio), were used in this study. CCLP1, HuCCT1, RBE, LX-2, and HCCC-9810 cells were cultured in Roswell Park Memorial Institute 1640 (RPMI-1640; catalog no. abs9484, univ-bio) medium or Dulbecco’s Modified Eagle Medium (catalog no. abs9483, univ-bio) supplemented with 10% fetal bovine serum (FBS; catalog no. SH30088.03HI, Cytiva) and 1% penicillin-streptomycin (catalog no. abs9244, univ-bio). HiBEpiC cells were maintained in epithelial cell culture medium supplemented with the recommended growth supplements according to the manufacturers’ instructions. All cells were cultured at 37 °C in a humidified incubator containing 5% CO_2_. Cells were routinely tested and confirmed to be free of mycoplasma contamination.

Mouse splenic CD8^+^ T cells and CD4^+^ T cells were isolated from mouse spleens. Briefly, spleens were harvested under sterile conditions and mechanically dissociated into single-cell suspensions. Red blood cells were lysed using red blood cell lysis buffer (catalog no. S0D9001, univ-bio), and splenocytes were filtered through a cell strainer. CD8^+^ T cells and CD4^+^ T cells were purified using magnetic bead-based selection kits (CD4^+^ T cells [catalog no. 19852, Stem cell]; CD8^+^ T cells [catalog no. 19858, Stem cell]) according to the manufacturers’ instructions. Purified T cells were cultured in RPMI-1640 medium supplemented with 10% FBS, 1% penicillin-streptomycin, 50 μmol/l β-mercaptoethanol (catalog no. abs9592, univ-bio), and recombinant mouse interleukin-2 (IL-2, 100 U/ml; catalog no. UA040171, univ-bio). When required, T cells were activated using anti-CD3/CD28 activation beads (2 μg/ml; catalog no. 11456D, Thermo Fisher). Cells were maintained at 37 °C in a humidified atmosphere with 5% CO_2_. For polarization, naïve CD4^+^ T cells (1 × 10^6^ cells/ml) were cultured with TGF-β (5 ng/ml; catalog no. HY-P70648, Medchemexpress), IL-2 (100 U/ml), and reduced anti-CD3/CD28 activation beads (0.5 μg/ml) for 3 to 5 days. CD4^+^CD25^+^Foxp3^+^ Tregs were quantified via flow cytometry (BD Biosciences). For naïve Tregs, cells were further purified using fluorescence-activated cell sorting.

Primary human and mouse macrophages were isolated from freshly resected ICC tumor tissues or paired adjacent nontumor tissues. Briefly, fresh tissue specimens were collected immediately after surgical resection and transferred to the laboratory in ice-cold sterile phosphate-buffered saline (PBS) (catalog no. SH30256, univ-bio) or RPMI-1640 medium supplemented with antibiotics. Necrotic areas, visible blood clots, and connective tissues were carefully removed. The tissues were then minced into small fragments using sterile scissors and digested in RPMI-1640 medium containing collagenase IV (1 mg/ml; catalog no. abs082456, univ-bio), collagenase II (1 mg/ml; catalog no. abs082505, univ-bio), DNase I (0.5 U/ml; catalog no. D8071, Solarbio), and hyaluronidase (0.5 mg/ml; catalog no. abs42225221, univ-bio) at 37 °C with gentle agitation. After enzymatic digestion, the cell suspension was filtered through a 70-μm cell strainer to remove undigested tissue debris and obtain a single-cell suspension. Red blood cells were lysed using red blood cell lysis buffer when necessary, followed by washing with PBS.

To enrich macrophages, the single-cell suspension was subjected to flow cytometry-based sorting. Human macrophages were isolated based on the expression of myeloid/macrophage markers, such as CD45, CD11B, and CD68 for human, and Cd11b and F4/80 for mouse (antibodies details can be find in Table S2), according to the experimental requirements and the manufacturers’ instructions. Alternatively, adherent macrophages were enriched by plating the single-cell suspension in complete RPMI-1640 medium and removing nonadherent cells after incubation. The isolated or enriched macrophages were cultured in RPMI-1640 medium supplemented with 10% FBS, 1% penicillin-streptomycin, and recombinant macrophage colony-stimulating factor (M-csf; 0.1 ng/ml, catalog no. abs04383, univ-bio) when required. Cells were maintained at 37 °C in a humidified incubator containing 5% CO_2_.

Mouse peripheral blood monocytes were isolated from freshly collected anticoagulated peripheral blood. After red blood cell lysis or density-gradient centrifugation, peripheral blood mononuclear cells were collected, washed, and resuspended in complete RPMI-1640 medium. Monocytes were enriched using a mouse monocyte isolation kit (catalog no. 19861, Stem cell). The isolated monocytes were cultured in RPMI-1640 medium supplemented with 10% FBS, 1% penicillin-streptomycin, and recombinant mouse M-csf at 37 °C in a humidified 5% CO_2_ incubator. The medium was refreshed every 2 to 3 days, and differentiated monocyte-derived macrophages were used after 5 to 7 days of culture.

The purity and viability of isolated macrophages were assessed by flow cytometry before downstream experiments. Cells with adequate viability were used for coculture assays, flow cytometric analysis, immunofluorescence staining, or other functional experiments.

### Single-cell suspension preparation and single-cell library preparation and sequencing

Fresh ICC tissues (human surgical specimens and murine liver tumor samples) were transported on ice to the laboratory, rinsed with prechilled PBS buffer to remove blood, and minced into 1- to 3-mm^3^ fragments. A combination of mechanical dissociation and enzymatic digestion was performed using a mixture of Collagenase II (1 mg/ml; catalog no. abs082505, univ-bio), Collagenase IV (1 mg/ml; catalog no. abs082456, univ-bio), hyaluronidase (0.5 mg/ml; univ-bio), and DNase I (0.5 U/ml; Solarbio), followed by 60-min oscillation incubation at 37 °C with intermittent pipetting to enhance dissociation. The enzymatic reaction was terminated by adding a culture medium (RPMI-1640, univ-bio), containing 10% FBS (Cytiva). The suspension was centrifuged (400 ×*g*, 5 min), and the supernatant was discarded. Undigested clumps were removed by sequential filtration through 100-μm cell strainers (catalog no. CSS025100, univ-bio) and 70-μm (catalog no. CSS0015040, univ-bio) cell strainers, followed by 2 to 3 PBS washes. Erythrocytes were lysed for 5 min, and debris was removed by centrifugation. After 2 additional PBS washes, cell density and viability (viability > 90%, nucleated cell rate > 90%) were assessed using an automated cell counter to meet scRNA-seq standards.

Single-cell suspensions were combined with Gel Beads in Emulsion (GEMs; 10x Genomics) containing oligonucleotides and encapsulated into oil droplets via microfluidic technology to achieve single-cell isolation. Cellular mRNA was captured by polyT-coated gel beads (10x Genomics), enabling cell-specific barcoding and unique molecular identifier (UMI; 10x Genomics) labeling. Reverse transcription within GEMs generated barcoded cDNA, which was amplified via PCR after emulsion disruption to construct full-length cDNA libraries. Fragmented cDNA was ligated with sequencing adapters (P5/P7) and sample indices to generate Illumina-compatible libraries. Library quality was verified by Qubit (10x Genomics), followed by paired-end sequencing on the Illumina platform (Beijing Novogene Biotechnology Co., Ltd.) using reference genomes GRCh38 (human) and Mm10 (mouse). All protocols adhered to the 10x Genomics platform guidelines.

### scRNA-seq data quality control

Raw scRNA-seq data were quantified using CellRanger (10x Genomics) and processed via the Seurat pipeline to create Seurat objects. Potential doublets were identified and removed using the DoubletFinder package (https://github.com/chris-mcginnis-ucsf/DoubletFinder). Cells with mitochondrial gene content exceeding 10% (Percent.mt score) were excluded. Quality thresholds were set as follows: 500 < nCount < 8,000 and nFeature > 300 per cell, yielding 264,155 cells for downstream analysis. TCR^+^ macrophages underwent additional validation to exclude residual doublets.

### scRNA-seq analysis pipeline

Post-quality control data were analyzed using the Seurat standard workflow. To address batch effects in sequencing data, batch correction was performed via the RunHarmony (Github) function, with correction parameters optimized based on the absence of batch-driven clustering in Uniform Manifold Approximation and Projection (UMAP) embeddings. High-dimensional features were visualized in 2-dimensional space using t-distributed Stochastic Neighbor Embedding (t-SNE) and UMAP. Cell clusters were annotated based on established lineage-specific markers: *KRT19*, *KRT8*, and *KRT9* for epithelial cells; *COL1A1*, *COL1A2*, and *PDGFRB* for fibroblasts; *PECAM1* and *VWF* for endothelial cells; *CD19*, *CD79A*, and *MS4A1* for B cells; *IGHG1*, *MZB1*, and *CD79A* for plasma cells; *CD68*, *CD11B*, *FCGR3B*, *CLEC9A*, *LYZ*, and *CD14* for myeloid cells; *CST3* and *CPA3* for mast cells; *CD3E*, *CD3D*, *CD8A*, *CD4*, *PRF1*, and *NKG7* for T/NK cells; *ALB* for hepatocytes; *HBA1*, *HBG1*, and *HBZ* for red blood cells (filtered and removed).

### Bulk RNA sequencing analysis

Tissue/cell samples were immediately flash-frozen in liquid nitrogen or preserved in RNAlater (catalog no. AM7021, Thermo Fisher) to prevent RNA degradation. Total RNA was extracted using column-based kits (catalog no. 587449, QIAGEN), with purity verified by Nanodrop (OD_260_/OD_280_ ≈ 2.0, OD_260_/OD_260_ > 1.8; Thermo Fisher) and integrity assessed via Agilent Bioanalyzer/Qubit (RIN > 7; Agilent Technologies). Polyadenylated eukaryotic mRNA was enriched using oligo (dT) magnetic beads (catalog no. 61005, Thermo Fisher), followed by fragmentation into 200- to 300-bp fragments. Double-stranded cDNA was synthesized through reverse transcription, end-repaired, and ligated with sequencing adapters (Illumina). Amplified libraries were validated for insert size (~300 bp peak) using Agilent 2100 (Agilent Technologies) and quantified by Qubit (Thermo Fisher). Paired-end sequencing (PE150) was performed on NovaSeq (Illumina) with a depth of 20 to 50 million reads per sample.

Raw reads were quality-checked by FastQC (Q30 > 80%; Babraham), with adapters and low-quality bases trimmed using Trimmomatic/Cutadapt (GitHub). Clean reads were aligned to the reference genome (GRCh38/mm10) via Hisat2, achieving > 70% alignment rates. Gene expression matrices were generated using HTSeq (Simon Anders), with data biases (5′/3′ preference) evaluated by RSeQC (GitHub). Differential gene expression analysis was performed using the limma package (Bioconductor). All technical parameters and methods related to bulk RNA-seq were provided by Beijing Novogene Biotechnology Co., Ltd.

### InferCNV analysis

The analysis pipeline integrated single-cell expression matrices, cell-type annotations (tumor vs. normal reference cells), and chromosomal gene coordinates. Fibroblast expression profiles served as the baseline to normalize tumor cell expression, mitigating confounding factors. Sliding genomic windows (~100 genes) were applied to compute expression deviations (amplification, elevated expression; deletion, reduced expression) relative to the baseline. Gaussian smoothing and median filtering were employed to suppress technical noise while retaining large-scale CNV signals. Hierarchical clustering of tumor cells based on CNV patterns revealed malignant subpopulations and clonal heterogeneity. Chromosomal heatmaps annotated with significant CNV regions were generated to validate malignant cells. For quantitative assessment, inferCNV scores were standardized across cells. Adjacent normal tissue-derived epithelial cells provided reference average inferCNV scores. Cluster-level inferCNV scores were then compared against this baseline to identify malignant subclusters. The detailed workflow of the inferCNV software can be found at https://github.com/broadinstitute/infercnv.

### Cell interaction analysis using CellphoneDB

The preprocessed single-cell expression matrix and cell-type annotations were imported into the CellphoneDB V4 Python module (GitHub). Ligand–receptor pairs coexpressed in >20% of cells within interacting cell types were systematically evaluated. Statistical significance (*P* value) was determined through permutation testing (1,000 iterations, random shuffling of cell labels). Pairs with *P* < 0.05 were retained for downstream analysis. Interaction networks were visualized using the ktplots package (Bioconductor), with edge weights representing interaction strength and node sizes reflecting cell-type abundance. The detailed workflow of the CellphoneDB software can be found at https://github.com/ventolab/CellphoneDB.

### PySCENIC analysis

The normalized scRNA-seq matrix (post-gene filtering) was analyzed through the following steps: (a) Coexpression network construction (GENIE3 algorithm identified transcription factor [TF]-target gene modules by inferring regulatory relationships from gene expression covariation). (b) Motif enrichment analysis (cisTarget database [hg38 motif annotations; https://resources.aertslab.org/cistarget/] scanned TF-binding motifs in evolutionarily conserved regulatory regions [phyloP ≥ 7]. TF-module pairs with significant motif enrichment [normalized enrichment score > 3.0] were retained). (c) Regulon activity scoring (the AUCell algorithm calculated cell-specific regulon activity scores [area under the curve, AUC] by ranking gene expression per cell. Thresholds [AUC > 0.2] identified subpopulations with activated TF networks). (d) Network visualization (TF activity matrices were plotted using ggplot2). A more detailed method came from pySCENIC V0.12.1 (GitHub).

### scRNA-seq trajectory analysis

Monocle2: Normalized expression matrix with highly variable genes. Nonlinear dimensionality reduction (DDRTree) mapped cells to a latent space, followed by minimum spanning tree (MST) computation. Assignment (Root state [monocytes]) was manually defined. Pseudotime values (0 to 1 scale) were calculated along MST branches. The detailed workflow of the Monocle2 software can be found at https://cole-trapnell-lab.github.io/monocle-release/.

ScVelo: ScVelo was used to estimate RNA velocity from unspliced and spliced mRNA counts quantified from aligned reads. RNA velocity was estimated using stochastic differential equations to model transcriptional dynamics. The velocity vectors were projected onto PAGA-derived UMAP embeddings to visualize cell-state trajectories. Streamlines indicated the direction of differentiation toward terminal states. The dynamical mode was used, with min_likelihood set to 0.2, corresponding to the default setting. The detailed workflow of the ScVelo software can be found at https://scvelo.readthedocs.io/en/stable/.

### Spatial transcriptomics sample preparation and analysis

Fresh ICC specimens were optimal cutting temperature-embedded, flash-frozen at −80 °C, and sectioned into 5 to 10 μm slices using a cryostat. Tissue sections were mounted onto spatially barcoded Visium slides (10x Genomics). Sections underwent protease K (10x Genomics) digestion with optimized permeabilization (30 min) to release RNA for polyT probe capture (10x Genomics). Reverse transcription generated spatially barcoded cDNA, which was amplified to construct sequencing libraries. Libraries were validated for insert size (~300 bp peak) via Agilent 2100 (Agilent Technologies), quantified by Qubit (Thermo Fisher), and sequenced on Illumina NovaSeq (PE150, Q30 > 85%; Illumina). Raw reads were quality-filtered using Trimmomatic (GitHub). Spatial barcode decoding and read alignment to reference genomes (GRCh38/mm10) were performed via Spaceranger (10x Genomics), generating a gene-spot expression matrix with spatial coordinates. Data normalization was performed via SCTransform (R package, Bioconductor) and unsupervised clustering was carried out using seurat (R package, Bioconductor), with spatial gene expression patterns visualized using the SpatialFeaturePlot function (R package, Bioconductor). All technical parameters and methods related to spatial transcriptomics were provided by Beijing Novogene Biotechnology Co., Ltd.

Expression quantification data were extracted from spatial transcriptomics analysis, followed by standardization of the data and UMAP dimensionality reduction analysis according to the standard seurat pipeline. After extracting the spatial coordinates of each spot in the spatial transcriptomics analysis, these spatial coordinates were replaced with the coordinate positions obtained after UMAP dimensionality reduction. Subsequently, we used the plot_density function from the Nebulosa-R package (Bioconductor) to plot the coexpression regions of 2 genes (parameter: adjust = 6), allowing us to roughly identify the spatial location of the cells.

### Macrophage M1/M2 polarization and TREM2^+^ macrophage scoring

Gene set enrichment analysis for macrophage subsets was used to assess macrophage characteristics. It was performed using AddModuleScore (R package, Bioconductor) and AUCell (rank threshold = 0.2, AUC threshold = 0.05; R package, Bioconductor) algorithms. Reference gene sets for M1 (pro-inflammatory), M2 (anti-inflammatory), and TREM2^+^ macrophages are detailed in Table S3. Methodological protocols are accessible via AddModuleScore (https://satijalab.org/seurat/reference/addmodulescore) and AUCell (https://github.com/aertslab/AUCell).

### Gene Ontology enrichment analysis

Differentially expressed genes (DEGs, *P* < 0.05) identified in prior analyses were subjected to Gene Ontology (GO) enrichment analysis using the clusterProfiler package (Bioconductor). Gene symbols were converted to ENTREZ IDs via org.Hs.eg.db (R package, Bioconductor), followed by enrichGO execution with parameters (keyType = “ENTREZID”), pAdjustMethod = “BH”, pvalueCutoff = 0.01. Significantly enriched terms (*P*.adjust < 0.05) were visualized via ggplot2 R package (Bioconductor), with dot plots displaying fold enrichment and term hierarchies.

### Scissor algorithm for prognosis-associated cell identification

The Scissor algorithm (method link [https://github.com/sunduanchen/Scissor]) was employed to identify macrophage subpopulations linked to ICC patient survival. Bulk RNA-seq data from 58 ICC patients with survival records served as the reference dataset, while our single-cell macrophage data (*n* = 7,142 cells) constituted the discovery dataset. Key parameters were optimized as follows: cutoff = 0.5 (top 10% phenotype-correlated cells retained), family = “Cox” (Cox proportional hazards regression), and alpha = 0.001 (regularization strength). This analysis identified 517 macrophages associated with poor prognosis. Protocol details can be found at https://github.com/sunduanchen/Scissor.

### DEG analysis

Single-cell differential expression was assessed using FindMarkers/FindAllMarkers R packages (default: logfc.threshold = 0.25, min.pct = 0.1, test.use = “Wilcoxon”; Bioconductor). The differential score (Diff) was calculated as: Diff = Pct.1 − Pct.2. Pct.1/Pct.2 denote the percentage of cells expressing a gene in cluster 1 vs. cluster 2. For bulk RNA-seq, limma R packages (Bioconductor) identified DEGs. All results were visualized via ggplot2 (geom_violin for single-cell scRNA-seq; geom_volcano for bulk RNA-seq).

### Survival analysis

Human survival curves were generated using the survival (Cox PH model) and survminer packages, excluding patients with ≤1-month follow-up or prior chemotherapy. The optimal expression cutoff for risk stratification was determined by surv_cutpoint (minprop = 0.2). Murine survival data were plotted in GraphPad Prism v9.0 (Kaplan–Meier method, log-rank test; Dotmatics).

### Cell subpopulation similarity analysis

For the cell subpopulation, similarity analysis was described in the main text. Briefly, mouse genes were converted to human orthologs using the mouse2human R package (Bioconductor). Human and mouse datasets were normalized and harmonized, followed by anchor point calculation via the FindTransferAnchors R package (with mouse data as the reference; Bioconductor). Predicted cell-population mappings were averaged to generate similarity scores, visualized as correlation heatmaps using the pheatmap R package (Bioconductor).

### Flow cytometry

Fresh tissue samples from tumor or adjacent tissues were rinsed twice with sterile prechilled PBS, minced, and transferred to 10-ml centrifuge tubes. Because of the dense texture of ICC tissues, mechanical dissociation combined with enzymatic digestion was performed using a solution containing Collagenase II (1 mg/ml; catalog no. abs082505, univ-bio), Collagenase IV (1 mg/ml; catalog no. abs082456, univ-bio), hyaluronidase (0.5 mg/ml; catalog no. abs42225221, univ-bio), and DNase I (0.5 U/ml; catalog no. D8071, Solarbio). Tissues were incubated at 37 °C with agitation (60 min for human samples; 30 min for murine tumors). Dissociated cells were filtered, subjected to erythrocyte lysis buffer (catalog no. R1010, Solarbio), and resuspended in chilled flow cytometry staining buffer (0.2 ml, final concentration 1 × 10^6^ cells/ml; catalog no. FC001, univ-bio). After Fc receptor blocking (15 min; catalog no. 88280S, univ-bio), cells were incubated with 2 μg of fluorescently labeled antibodies at 4 °C for 40 min, washed with PBS, and filtered through nylon mesh (catalog no. 4436TC, univ-bio) before acquisition. Flow cytometry data were analyzed using FlowJo V10.8.1 (BD Biosciences): single cells were gated by Forward Scatter Area (FSC-A) vs. Forward Scatter Height (FSC-H), dead cells were excluded via viability dye staining (0.1 μg/1 ml; catalog no. 564406, BD Biosciences), and target populations were quantified after compensation matrix calibration. The antibodies used in this study are summarized in Table S2.

### mIF and IHC

Fresh tissues collected from human and mouse ICC tumor were fixed in 4% paraformaldehyde (catalog no. E-IR-R113, univ-bio) for 48 h, rinsed with PBS, dehydrated through a graded ethanol series (70%, 80%, 90%, 95%, and 100% ethanol), cleared with xylene (twice, 15 min each), and embedded in paraffin through 3 wax immersion steps (15 min initial, 40 min × 2) at 55 °C. Tissue blocks were sectioned at 6 μm thickness, floated on 40 °C water, mounted on slides, and baked overnight at 60 °C. For antigen retrieval, slides were microwaved in EDTA buffer (pH 8, 0.5 mmol/l, catalog no. 15575020, univ-bio), blocked with 5% BSA (catalog no. abs9156, univ-bio), and incubated with primary antibodies in a humidified chamber at 4 °C overnight. Secondary antibodies and 4′,6-diamidino-2-phenylindole (catalog no. sc-3598, univ-bio) were sequentially applied, followed by anti-fade mounting with anti-fade mounting medium (catalog no. abs090692, univ-bio). Slides were scanned using a high-resolution fluorescence scanner (Pannoramic MIDI) and analyzed with slideViewer V2.6.0 software (3DHISTECH) for colocalization and quantitative signal assessment. The antibodies used in mIF were summarized in Table S2.

For IHC, paraffin-embedded tissue sections were deparaffinized, rehydrated, and subjected to antigen retrieval using EDTA buffer. After blocking endogenous activity and nonspecific binding, the sections were incubated with primary antibodies overnight at 4 °C. The next day, sections were incubated with appropriate secondary antibodies, followed by chromogenic detection. Finally, the slides were counter-stained, dehydrated, mounted, and imaged under a microscope for subsequent analysis. The antibodies used in IHC are summarized in Table S2.

Criteria for determining coenrichment of TREM2 and POSTN at the tumor boundary: When selecting fluorophores for TREM2 and POSTN staining, we intentionally chose those with minimal spectral overlap. The acquired images were then processed in ImageJ (National Institutes of Health) for cropping and region-of-interest (ROI) delineation. The ROI was defined as the area within 200 μm from the tumor–normal boundary toward the tumor side. Within each ROI, background signal was removed, and the TREM2 and POSTN channels were separated. The Pearson correlation coefficient between the 2 channels was subsequently calculated using the Scatter J (https://download.savannah.gnu.org/releases/scatterj/) plugin. The calculated Pearson correlation coefficients for each ROI are provided in Table S4. A Pearson correlation coefficient > 0.5 was considered indicative of colocalization, while values below this threshold were regarded as noncolocalized.

### Enzyme-linked immunosorbent assay

TREM2^+^, TREM2^−^, and TCR^+^ macrophages isolated via flow cytometry were stabilized and cultured in a serum-free medium (RPMI-1640). After 24 h, supernatants were collected and filtered through 0.22-μm membranes (catalog no. abs930612DE, univ-bio). Cytokine levels were measured using enzyme-linked immunosorbent assay (ELISA) kits (GZMA [catalog no. MBS2232, Thermo Fisher]; PLAU/uPA [catalog no. E-EL-H1779, elabscience]) according to manufacturer protocols. Optical density values were corrected by subtracting blank control values. Standard curves were generated in excel. Sample concentrations were calculated based on the curve and dilution factors. Data were analyzed in GraphPad Prism V9.0 (Dotmatics): 2-group comparisons used Student *t* test, while multigroup comparisons employed one-way analysis of variance (ANOVA) with post hoc pairwise testing.

### Western blotting

Cells at 90% confluency were washed with PBS and dissociated using 0.25% trypsin-EDTA (37 °C, 2 min; catalog no. 25200056, Thermo Fisher). The reaction was neutralized with 5 ml of medium (with 10% FBS). Cell pellets were lysed in ice-cold RIPA buffer (catalog no. P0013B, Beyotime) containing 1% phenylmethyl-sulfonyl fluoride (catalog no. SH0906, Beyotime), sonicated for 10 min, and centrifuged to collect supernatants. Protein concentrations were quantified via BCA assay using the BCA protein assay kit (catalog no. P0012, Beyotime). Denatured proteins (heated at 100 °C for 5 min using a metal bath heater) were separated by sodium dodecyl sulfate–polyacrylamide gel electrophoresis (catalog no. P0012A, Beyotime), transferred to polyvinylidene fluoride membranes (catalog no. FFP101, Beyotime), and blocked with Western protein-free rapid blocking buffer (catalog no. P0213, Beyotime). Membranes were incubated with primary antibodies overnight at 4 °C, followed by species-matched horseradish peroxidase-conjugated secondary antibodies (1 h, room temperature; catalog no. SH3282, Beyotime). After tris-buffered saline with Tween-20 (catalog no. ST673, Beyotime) washes, signals were detected using a dual-color infrared imaging system (Tanon), and band intensities were quantified. The antibodies used in Western blotting are summarized in Table S2.

### Coculture system

Flow cytometry-sorted macrophages were counted and seeded into Transwell inserts (5 × 10^4^ cells/insert, 200 μl of RPMI-1640 with 10% FBS). The lower chamber contained HuCCT1 cells (1 × 10^5^ cells/well) or CD8^+^/CD4^+^ T cells in 600 μl of medium. For tumor cell migration/invasion assays, HuCCT1 cells were harvested after 24 to 30 h. T cells were cultured without IL-2 replenishment during medium changes every 24 h. Cocultures were terminated at 48 to 96 h for downstream analysis.

### Wound healing assay

Twelve-well plates were premarked with horizontal lines on the back. Log-phase cells were seeded into the plates and cultured overnight to achieve 85% confluency. After 12 to 16 h, the medium was replaced with basal medium (RPMI-1640) for an additional 12-h incubation to reach 100% confluency. A 200-μl pipette tip was used to create uniform scratches parallel or perpendicular to the predrawn lines. Cells were washed 3 times with PBS to remove debris and cultured in a 2% serum medium (RPMI-1640) for 24 h to minimize cell proliferation during the wound-healing assay. This low-serum condition was used to reduce the contribution of proliferation to wound closure, allowing the assay to mainly reflect cell migration. Images were captured under a microscope after PBS washes. Migration rates (wound healing rates) were calculated using ImageJ (NIH): migration rate (%) = (initial scratch area − final scratch area)/initial scratch area × 100. Data were analyzed in GraphPad Prism V9.0 (Dotmatics) via one-way ANOVA with pairwise comparisons.

### Transwell migration and invasion assay

Cells were starved in RPMI-1640 for 16 to 20 h at 80% confluency. For invasion assays, Transwell inserts were precoated with Matrigel (migration assays omitted this step; catalog no. C0372, Beyotime). Pre-chilled Matrigel was diluted with serum-free medium (64 μl of medium plus 60 μl of Matrigel), added to Transwell inserts, and incubated at 37 °C for 3 h to polymerize. The lower chamber was filled with 600 μl of RPMI-1640 containing 20% FBS. Starved cells (5 × 10^4^ Hccc-9810 cells/insert in 200 μl of serum-free medium) were seeded into the upper chamber. After incubation (migration 24 h and invasion 30 h for HuCCT1; migration 24 h and invasion 48 h for Hccc-9810), cells were fixed with 4% paraformaldehyde (20 to 30 min; catalog no. P0099, Beyotime) and stained with 1% crystal violet (10 min; catalog no. C0121, Beyotime). Nonmigrated/invaded cells were removed with a cotton swab. Migrated/invaded cells in 5 random fields were counted under a microscope (10×; Zeiss) and analyzed using ImageJ 1.54 (National Institutes of Health). Statistical significance was assessed via one-way ANOVA.

### CD8^+^ T cell cytotoxicity assay

CD8^+^ T cells isolated from OT1 mouse (catalog no. T059617, GemPharmatech) spleens were expanded as described above. Murine ICC cells were transduced with ovalbumin (OVA)-encoding lentivirus (according to manufacturer protocol), followed by 3 to 4 rounds of antibiotic selection. OVA expression was validated by ELISA (catalog no. ab289830, Abcam) before coculture. Expanded OT1-CD8^+^ T cells were first indirectly cocultured with macrophages for 48 h. Tumor cells and CD8^+^ T cells were then cocultured at a 1:1 ratio in adherent plates. After 48 h, plates were gently washed with PBS (3 times) to remove debris. The remaining tumor cells were quantified via automated microscopy (Celigo Image Cytometer; DAKEWE) using confluence analysis.

### ICC mice model construction

Male C57BL/6J mice (5 to 6 weeks, weight 19 to 20 g; catalog no. SM-001, Shanghai Model Organisms Center) and *Trem2*-KO mice (5 to 6 weeks, weight 19 to 20 g; catalog no. NM-KO-190402, Shanghai Model Organisms Center) were purchased from the Shanghai Model Organisms Center. For Yap-Akt model, a plasmid cocktail containing pT3-EF1α-HA-myr-Akt (15 μg; catalog no. 31789, Addgene), pT3-EF1α-YAPS127A (30 μg; catalog no. 46049, Addgene), and Sleeping Beauty transposase 100 (SB100, 2 μg; catalog no. 34879, Addgene) in 2 ml of saline (catalog no. ST341, Beyotime) was delivered via hydrodynamic tail vein injection. Liver tumorigenesis was initiated within 7 days. For the Kras-P19 model, plasmids pCaggs-KRAS^G12D^ (20 μg; provided by Prof. Lei Wang of Nanjing Drum Tower Hospital), CRISPR/Cas9 sg-p19 (pX330-sg-p19, 15 μg; provided by Prof. Lei Wang of Nanjing Drum Tower Hospital), and SB100 (3 μg; catalog no. 34879, Addgene) in 2 ml of saline were hydrodynamically injected. Tumor onset occurred at ~18 days. All mice were housed in a specific pathogen-free animal facility under controlled environment. At the end of the experiment or when humane endpoints were reached, mice were deeply anesthetized with an approved anesthetic agent. After confirming deep anesthesia by the absence of pedal reflex, mice were euthanized by cervical dislocation. Death was confirmed by the absence of respiration, heartbeat, and response to external stimuli before sample collection. Humane endpoints were established to minimize animal suffering. Mice were euthanized if they exhibited any of the following conditions: severe weight loss, persistent anorexia or dehydration, obvious lethargy or inability to move normally, severe ulceration or necrosis at the tumor site, signs of pain or distress that could not be relieved, or impaired respiration. For tumor-bearing mice, euthanasia was also performed when the tumor volume reached the maximum size permitted by the institutional animal care guidelines, or when the tumor interfered with normal movement, feeding, or drinking. Animals reaching any humane endpoint were immediately removed from the study and euthanized humanely.

### ICC mouse treatment and survival analysis

Tumor burden was assessed by in vivo bioluminescence imaging using an IVIS Spectrum system before mice were randomly assigned to treatment groups. In the Kras-P19 induced ICC mouse model, treatment was initiated on day 20 after model establishment. In the Yap-Akt induced ICC model, treatment was initiated on day 8 after model establishment.

Mice received intraperitoneal injections of 200 μl per dose every 3 days for a total of 6 doses. Anti-Csf1r antibody (catalog no. BE0273, BioXcell), anti-Pd-1 antibody (catalog no. BE0213, BioXcell), anti-Trem2 antibody (catalog no. BE0447, BioXcell), and anti-Timd4 antibody (catalog no. BE0171, BioXcell) were administered at doses of 200, 150, 200, and 200 μg per injection, respectively. Mice in the control group received 150 μg of isotype antibody per injection.

Method for generating AAV-mediated *Trem2* knockdown mice: Viral stock containing 5 × 10^11^ vector genomes was diluted in 0.2 ml of saline and then administered to mice via tail vein injection using a 1-ml syringe. The control group was injected, following the same procedure and at the same viral titer, with an AAV carrying a scrambled sequence. The AAV vectors were obtained from Weizhen Biosciences, and the relevant sequences are provided in Table S5.

For survival analysis, mice were treated according to the same schedule and dosages described above. To maintain therapeutic exposure, mice that remained alive after the initial 6 doses received additional treatment every 3 days.

### Preparation of fibroblast activation protein-targeted Mal-DSPE-PEG2000-modified pirfenidone-loaded liposomes (FAP-MDP-Pirfen)

Egg yolk phosphatidylcholine (100 mg/l, Egg-PC; catalog no. 163-21181, Beyotime), cholesterol (10 mg/l; catalog no. ST1155, Beyotime), and Mal-DSPE-PEG2000 (12.5 mg/ml; catalog no. HY-140739, Medchemexpress) were dissolved in 100 μl of ethanol as the organic phase. Pirfenidone (0.2 mg/ml; catalog no. HY-B0673, Medchemexpress) was dissolved in 10 μl of dimethyl sulfoxide (catalog no. HY-Y0320C, Medchemexpress), and the 2 solutions were thoroughly mixed under ultrasonication. The mixture was then slowly injected into 890 μl of ultrapure water, followed by stirring at room temperature for 10 min to disperse the lipids. The resulting liposome suspension was centrifuged using an ultracentrifuge (100,000 ×*g*, 20 min). The supernatant was discarded, and the pellet was resuspended in 1 ml of ultrapure water. This centrifugation and resuspension process were repeated 3 times to completely remove organic solvents, ultimately yielding pirfenidone-encapsulated liposomes.

For antibody-conjugated liposomes, the prepared liposomes were incubated overnight at 4 °C in PBS with reduced anti-Fap antibodies (catalog no. HY-P990245, Medchemexpress). The particle size and polydispersity index of the liposomes were determined by dynamic light scattering (Zetasizer Nano ZSP, Malvern Instruments). The encapsulation efficiency of pirfenidone was analyzed using high-performance liquid chromatography (Thermo Fisher). The concentration of antibodies conjugated to the liposome surface was quantified by micro-BCA protein assay.

### Swimming plot

To compare changes in the relative abundance of cell subtypes between type 1 and type 2, the proportion of each target cell population among total cells was first calculated for each sample. The central tendency of the proportions for each cell population in the type 1 and type 2 groups was then determined mean, and the relative change was calculated as follows:

Ratios of cell types in total cells from type 1 versus type 2. The ratio was calculated as follows: $\text{Ratio}=\frac{\text{proportion of target cells among total cells in type}\;1\;}{\text{proportion of target cells among total cells in type}\;2}$ − 1.

A relative-change value greater than zero indicates an increased relative abundance in type 1 compared with type 2, whereas a value less than zero indicates a decreased relative abundance. Results were visualized using lollipop plots, in which each dot represents the relative change for an individual cell population and zero denotes no change. Between-group differences in cell proportions were assessed using a 2-sided Mann–Whitney *U* test.

### Statistical analysis

Statistical analyses were performed using GraphPad Prism version 9.0 and R software. Data visualization was conducted using GraphPad Prism V9 and R packages including sjPlot, ggplot2, survival, and survminer. Clinicopathological characteristics between marker-high and marker-low groups were analyzed using the χ^2^ test or Fisher’s exact test. Related clinical association tables and plots were generated using the sjPlot package in R. Kaplan–Meier survival curves were used to evaluate overall survival, and differences between groups were compared using the log-rank test. Comparisons between 2 groups were performed using Student *t* test, or Mann–Whitney *U* test, depending on the experimental design and data distribution. Multiple-group comparisons were performed using one-way ANOVA followed by Tukey’s or Dunnett’s post hoc test, or by the Kruskal–Wallis test for nonparametric data. Two-way ANOVA was used for grouped or time-course comparisons where appropriate.

For immunohistochemical analyses, H-scores were compared using one-way ANOVA followed by appropriate post hoc tests, and associations with clinicopathological features were analyzed using χ^2^ tests. Flow cytometry and mIF quantifications were analyzed using paired *t* test, Mann–Whitney *U* test, or ANOVA as appropriate.

Differences in cell subtype distribution between tissue groups were analyzed using the Mann–Whitney *U* test. Gene expression data were normalized by TPM transformation followed by log^2^ normalization where indicated.

All statistical tests were 2-sided. Data are shown as mean ± SD unless otherwise stated. *P* < 0.05 was considered statistically significant. Significance levels are indicated as *P < 0.05; **P < 0.001; ***P < 0.001; ****P < 0.0001.

## Results

### Study design

To comprehensively characterize macrophage heterogeneity in ICC, we performed scRNA-seq on tumor tissues and peritumoral tissues from 16 untreated ICC patients pathologically confirmed in our institutional cohort (in-house scRNA-seq human cohort, Table S6). To enhance the robustness of the results, we integrated publicly available scRNA-seq data from 8 additional ICC cases (GSE138709, Table S6). Detailed characteristics of all patients are summarized in Table S1. After stringent quality control to remove cells with mitochondrial gene content exceeding 15% and doublets identified using DoubleFinder, we retained 320,143 high-quality cells, including 18,677 macrophages. To validate findings from human cohorts, we analyzed scRNA-seq data from tumor tissues of 2 ICC mouse models: (a) Kras-P19-induced ICC mice (in-house scRNA-seq kras-mice cohort, Table S6) sampled at 3, 6, and 8 weeks postinduction (*n* = 3 per time point), and (b) Yap-Akt-induced ICC mice (in-house scRNA-seq yap-mice cohort, Table S6) harvested at 7,14, and 28 days postinduction (*n* = 3 per time point). Additionally, we performed scRNA-seq on *Trem2*-knockout (KO, in-house scRNA-seq kras-mice-Trem2KO cohort, Table S6) mice with Kras-P19-induced ICC at 8 weeks postinduction. Detailed information of these datasets are summarized in Table S6.

Clinical cohort validation was conducted through IHC and mIF on tumor tissues from 39 ICC patients and tumor border regions from 23 cases (Table S1) to assess macrophage distribution and immune barrier architecture (Fig. 1A). Bulk RNA-seq data from 105 ICC patients (EHBH-ICC cohort, Table S6) with paired tumor and adjacent nontumor tissues further complemented our multi-omics approach, providing crucial insights into the role of macrophage in tumor ecosystems.

**Fig. 1.**
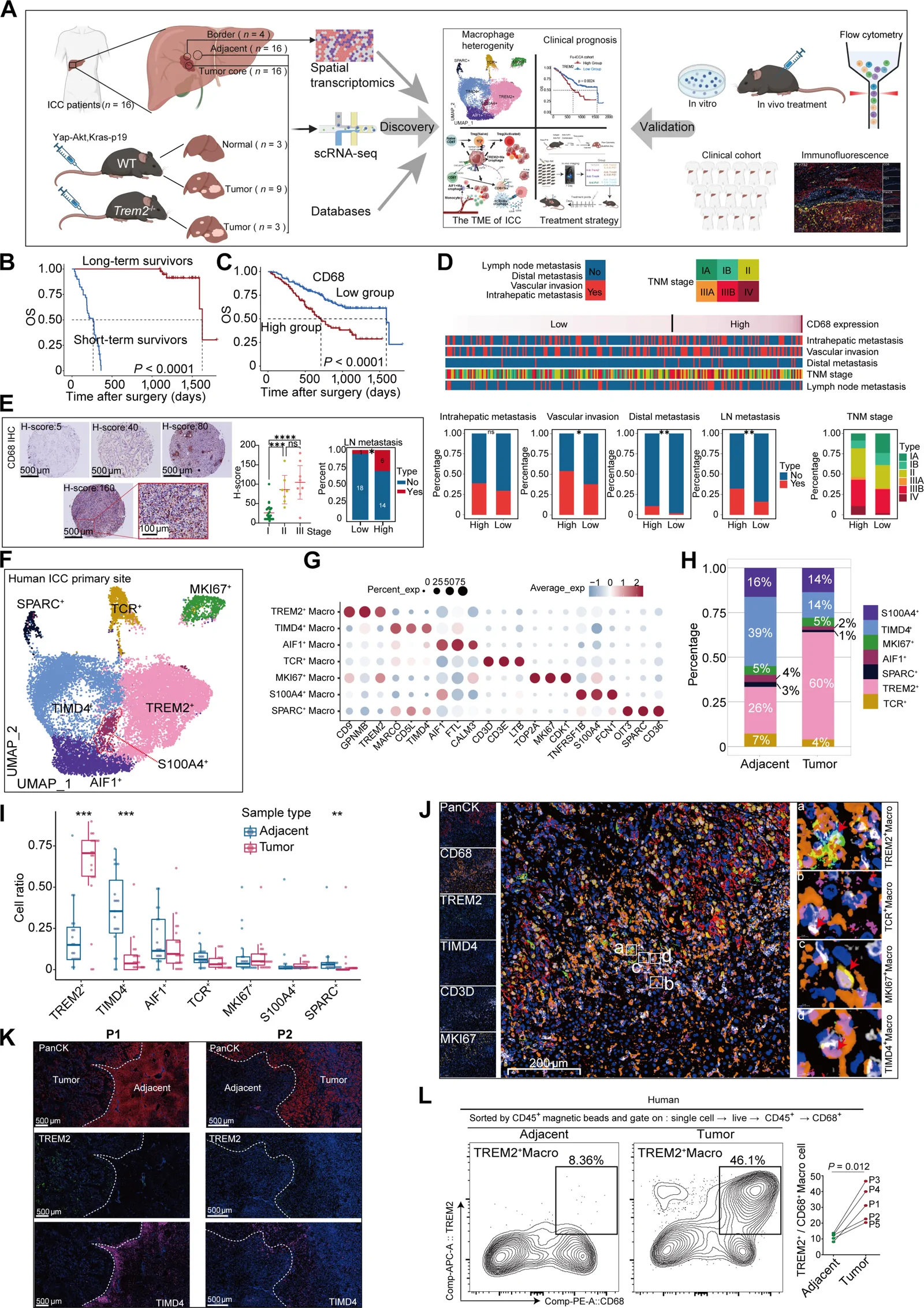
Macrophage abundance is associated with clinical outcome and reveals distinct macrophage subsets and spatial distributions in ICC. (A) Workflow of this study. (B) Kaplan–Meier overall-survival analysis comparing long-term and short-term survivors in the FuDan-ICC cohort. Survival curves were compared using the log-rank test. (C) Kaplan–Meier overall-survival analysis comparing patients with high and low CD68 expression in the FuDan-ICC cohort. Survival curves were compared using the log-rank test. (D) Clinicopathological characteristics between CD68-high and CD68-low groups in the FuDan-ICC cohort (*P < 0.05, χ^2^ test). The incidence rates of vascular invasion, distant metastasis, and LN metastasis, intrahepatic metastasis, and TNM stage in ICC patients increased with rising CD68 expression levels. (E) CD68 immunohistochemical staining of tissue microarrays from 39 ICC tumor tissues (stage I, *n* = 83; stage II, *n* = 81; stage III, *n* = 85; stage IV, *n* = 13), with H-score evaluation and association with clinicopathological characteristics (^***^P < 0.001; ^****^P < 0.0001; ns, not significant; Kruskal–Wallis test followed by Dunn’s multiple-comparisons test). (F) UMAP plot showed macrophage subtypes identified by scRNA-seq analysis of human ICC primary and adjacent tissues. (G) Dot plot showed representative marker genes of distinct macrophage subtypes identified by scRNA-seq analysis. Dot size indicates the percentage of cells expressing the indicated gene, and color intensity indicates the average normalized expression level. (H) Relative distribution of macrophage subsets in adjacent and tumor tissues, shown as the percentage of cells assigned to each macrophage subset. (I) Comparison of macrophage-subset proportions between paired adjacent and tumor tissues. Each point represents one sample. Exact significance levels are indicated in the figure (^**^P < 0.01; ^***^P < 0.001; Paired comparisons–Wilcoxon signed-rank test). (J) Multiplex immunofluorescence (mIF) images showing the spatial distribution of macrophage subsets in ICC tissues. The colored arrows indicate representative cells or regions corresponding to the indicated macrophage subsets. (K) Representative mIF images showing the tissue distribution of TREM2 and TIMD4 in ICC specimens. Tumor and adjacent regions are separated by the dashed line. (L) Flow cytometry analysis of TREM2^+^ macrophage proportions in paired tumor and adjacent tissues from ICC patients (*n* = 5, Paired comparisons–Wilcoxon signed-rank test). Abbreviations: ICC, intrahepatic cholangiocarcinoma; UMAP, Uniform Manifold Approximation and Projection; scRNA-seq, single-cell RNA sequencing; mIF, multiplex immunofluorescence; H-score, histological score; LN, lymph node; TNM, tumor-node-metastasis; OS, overall survival.

### Macrophage infiltration is a critical prognostic factor in ICC

To elucidate the relationship between macrophages and the clinical characteristics of ICC patients, we conducted a survival analysis on a subset of patients from the FuDan-ICC cohort (excluded those who had undergone prior treatment; Table S6) [21]. Patients were categorized into long-term survivors (overall survival more than 36 months) and short-term survivors (overall survival between 1 and 24 months; Fig. 1B). DEG analysis between these groups revealed that short-term survivors exhibited higher expression of genes associated with tumor progression and tumor markers, such as S100P, PLAUR, AGR2, MUC1, and CEA (Fig. S1A), as well as markers of macrophages (CD68 and MARCO) and fibroblasts (POSTN; Fig. S1A). Notably, the expression levels of macrophage-specific genes, such as CD68, MARCO, CD9, CAPG, and TREM2, were associated with higher risk scores (Fig. S1B).

GO enrichment analysis of the up-regulated genes in short-term survivors showed significant enrichment of terms related to epithelial cell characteristics (Fig. S1C) and myeloid cell features, particularly those associated with monocytes, highlighting their importance in ICC prognosis (Fig. S1D). Subsequent scRNA-seq analysis showed that the expression levels of neutrophil (FCGR3B), monocyte (FCN1), and dendritic cell markers (CLEC9A and CD1C) were associated with prognosis (Fig. S1E and F). Consistent with previous reports, our results demonstrated that dendritic cells were associated with a favorable prognosis in ICC [22], whereas monocytes, macrophages [23], and neutrophils [24] were associated with poor prognosis (Fig. 1C and Fig. S1G).

Further analysis revealed significant associations between macrophage levels and ICC patient characteristics, such as metastasis, vascular invasion, lymph node metastasis, and tumor clinical stage (Fig. 1D). Additionally, we performed CD68 (a macrophage marker) immunohistochemical staining on tumor tissues from 39 patients with ICC and demonstrated that higher CD68 expression was associated with more advanced clinical stage and a higher rate of lymph node metastasis in patients with ICC (Fig. 1E), consistent with the findings of Ruffolo et al. [23]. Given the poor response of ICC to immunotherapy and the major role of CD8^+^ T cells as effector cells [4], we screened the cell types most correlated with exhausted CD8^+^ T cells in the FuDan-ICC cohort and EHBH-ICC cohort. In both cohorts, genes that showed the strongest positive correlations with the CD8^+^ T cell exhaustion score were predominantly associated with myeloid and macrophage cells, including C1QB, C1QC, MS4A6A, CSF1R, TNFSF13B, and SAMHD1 (Fig. S1H and I). Consistently, GO enrichment analysis of these positively correlated genes highlighted biological processes related to myeloid and macrophage migration, activation, and differentiation (Fig. S1J and K). UMAP visualization further showed that representative positively correlated genes were preferentially expressed in macrophage and myeloid cell clusters (Fig. S1E and L). These results indicated a close association between macrophage-enriched transcriptional programs and CD8^+^ T cell exhaustion, suggesting that myeloid cells, especially macrophages, may contribute to the exhausted CD8^+^ T cell phenotype in ICC.

Moreover, we observed significantly elevated macrophage levels in patients with *KRAS* and *TP53* mutations, with a similar trend in *IDH1*-mutant patients (Fig. S1M). These mutations directly or indirectly regulate the monocyte–macrophage through complex mechanisms. For instance, *KRAS* mutations in colorectal cancer [25] positively correlate with TAM presence. In esophageal squamous cell carcinoma, colony-stimulating factor-1 (CSF1) expression is significantly up-regulated in a p53-R172H-dependent manner, further promoting monocyte–macrophage expansion [26]. In cholangiocarcinoma, *IDH1* mutations directly increase M2-like TAMs [27].

Given the current challenges in ICC treatment, we hypothesized that macrophages may be a key factor contributing to the poor efficacy of immunotherapy in ICC, particularly in patients with *TP53* or *KRAS* mutations. The role of macrophages in reshaping the TME may be especially pronounced in these cases.

### Heterogeneity of macrophages in ICC

To illustrate the heterogeneity of macrophages in ICC, we integrated scRNA-seq data generated from tumor and adjacent nontumor tissues of 16 ICC patients (in-house scRNA-seq human cohort, Table S6), together with ICC tumor and adjacent nontumor tissue data from the public database GSE138709 (Table S6). In total, the dataset comprised 19 adjacent nontumor tissues and 21 tumor tissues. Detailed sample information is provided in Table S1. Macrophages were classified into 7 cell clusters (Fig. 1F and G), all of which expressed of CD68 (Fig. S2A). Among these, TREM2^+^ macrophages were predominantly enriched in tumor tissues, whereas TIMD4^+^ macrophages were more abundant in adjacent nontumor tissues (Fig. 1H). Although SPARC^+^ macrophages also showed a significant difference between adjacent tissues and tumors, their cell numbers were relatively low (Fig. 1I). The distributions of other macrophage subtypes showed no significant differences between tumor and adjacent tissues. Notably, TREM2^+^ macrophages represented the predominant population within tumor tissues (Fig. 1I). Consistent with previous studies [28–30], TREM2^+^ macrophages have been implicated in tumor progression. Moreover *Trem2*-deficient mice exhibit greater resistance to the growth of various cancers than wild-type mice. In addition, TREM2^+^ macrophages highly expressed CD9 and GPNMB, whereas TIMD4^+^ macrophages exhibited elevated levels of MARCO and CD5L (Fig. 1G). Prior research suggests that TIMD4^+^ macrophages may represent Kupffer cells [31–33]. Beyond these 2 spatially distinct subsets, we identified several unique macrophage cell subtypes. For instance, a macrophage subtype expresses T cell-associated markers (CD3D, CD3E, and LTB; Fig. 1G), the functional implications of which remain unclear. To our knowledge, only one study has described that monocyte-derived macrophages carry T cell receptors (TCRs) and can accumulate in caseous granuloma [34]. scRNA-seq data have revealed that this subtype showed high expression of FYN, LCK, and LAT (Fig. S2A), representative TCR-related genes [35]. Compared with other subtypes, TCR^+^ macrophages also display high expression levels of CD8^+^ T cell markers, including CD3E, IFNG, GZMA, and GZMB (Fig. S2B). Subsequent CD68^+^CD3^+^ cells (TCR^+^ macrophages), CD68^+^CD3^-^ cells (TCR^-^ macrophages), and effector CD8^+^ T cells (CD8^+^CD44^+^ T cells) were sorted by flow cytometry. ELISA analysis confirmed high extracellular levels of GZMA released by these cells (Fig. S2C and D). Additionally, we further validated the presence of TCR^+^ macrophages using Cite-seq technology (Fig. S2E). More importantly, TCR^+^ macrophages may be directly involved in tumor-cell killing. Additionally, marker genes associated with macrophage subtypes in human ICC are presented in greater detail in Table S7.

To further validate the presence and spatial distribution of various macrophage subtypes, we conducted mIF staining on tumor and adjacent tissues from ICC patients, showing that TREM2^+^ macrophages were predominantly localized in tumor and peritumor tissues, whereas TIMD4^+^ macrophages were mainly detected in peritumor and adjacent nontumor tissues (Fig. 1J and K). TCR^+^ macrophages and MKI67^+^ macrophages were present in the tumor, peritumor, and adjacent regions (Fig. 1J). Flow cytometry of paired tumor and adjacent tissues further confirmed the enrichment of TREM2^+^ macrophages in tumor regions (Fig. 1L). Spatial transcriptomics further demonstrated the abundance of TREM2^+^ macrophages in the peritumoral region (Fig. S2F). Collectively, these macrophage subtypes were differentially distributed in the TME, underscoring their distinct roles in tumor progression and immune modulation.

### Transcriptional similarity of macrophage subtypes between humans and mice with ICC

To validate the stability of macrophage subtypes identified from single-cell transcriptomic data, we reclustered these macrophages based on TF activity profiles. Consistently, the macrophages from human ICC tissues were also partitioned into 7 clusters (Fig. S3A). The TREM2^+^ macrophage cluster showed substantial overlap with cluster 0, including high VDR and PPARD activity (Fig. S3B and C). Given that VDR and PPARD are critical regulators of fatty acid metabolism [36], our findings suggest that TREM2^+^ macrophages may be activated by fatty acids or their metabolites. Interestingly, TCR^+^ macrophages showed considerably higher eomesodermin (EOMES) activity (Fig. S3B). EOMES is a critical TF mediating the effector functions of CD8^+^ T cells [37], raising the possibility that TCR^+^ macrophages exhibit a T cell-like transcriptional program. Additionally, the subtypes characterized by TIMD4, AIF1, MKI67, and TCR also exhibited stable TF activity patterns (Fig. S3B).

To examine cross-species conservation, we analyzed 2 mouse models of ICC. In Yap-Akt-induced ICC mice, we identified macrophage subtypes with expression patterns similar to those observed in human ICC, including Trem2^+^, Mki67^+^, and Timd4^+^ populations (Fig. 2A and B). In Kras-P19-induced ICC mice, Trem2 did not exclusively mark a single cluster, but subtypes such as Il1r2^+^ macrophages and Arg1^+^ macrophages exhibited high expression of Trem2 (Fig. 2C and D and Fig. S3D). Furthermore, in an independent dataset (GSE171443; Table S6), we observed significantly increased Trem2 expression in tumor tissues compared with normal liver tissues from control mice, with the highest expression observed in the mice with Kras-P19-induced ICC (Fig. 2E). Conversely, Timd4 expression was markedly reduced in Kras-, Yap-, and Nicd-induced tumor tissues of ICC mice (Fig. 2F). These findings suggest that Trem2 is overexpressed during tumorigenesis and indicative of the level of TAMs. In many studies, TIMD4 and CD5L have been recognized as characteristic markers of tissue-resident macrophages [17,38,39]. In the liver, tissue-resident macrophages are referred to as Kupffer cells. Consistently, in both mouse models of ICC, the proportion of Trem2^+^ macrophages increased, whereas that of Timd4^+^ macrophages decreased during tumor progression (Fig. 2G and H).

**Fig. 2.**
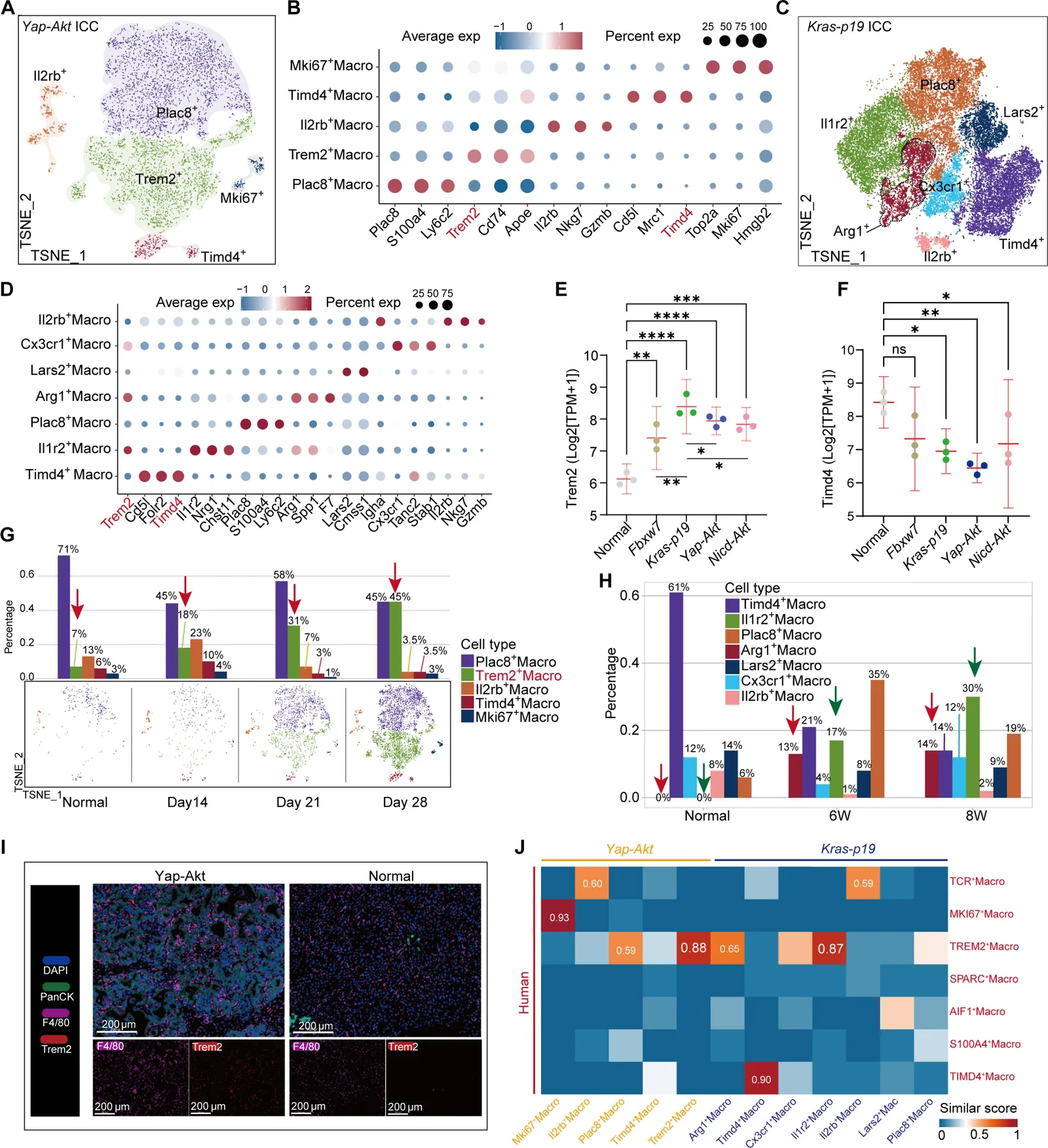
Conservation of macrophage cell subtypes across species in the ICC microenvironment. (A) t-SNE plot showing macrophage subclusters in Yap-Akt-induced ICC mice. (B) Dot plot showing representative marker genes used to annotate macrophage cell subtypes in Yap-Akt-induced ICC mice. Dot size indicates the percentage of cells expressing the indicated gene, and color intensity represents the average normalized expression level. (C) t-SNE plot showing macrophage subclusters in Kras-p19-induced ICC mice. (D) Dot plot showing representative marker genes used to annotate macrophage cell subtypes in Kras-P19-induced ICC mice. Dot size indicates the percentage of cells expressing the indicated gene, and color intensity represents the average normalized expression level. (E and F) Trem2 (E) and Timd4 (F) expression levels in ICC mice (Fbxw7, Yap-Akt, Kras-p19, and Nicd-Akt) versus normal liver (expression level following TPM transformation and log2 normalization, the results indicated that compared with normal mouse liver tissue; ^*^P < 0.05, ^**^P < 0.01, ^***^P < 0.001, ^****^P < 0.0001 by one-way ANOVA followed by Dunnett’s tests). (G and H) Temporal changes in the composition of macrophage cell subtypes in Yap-Akt-induced ICC mice (G) and Kras-P19-induced ICC mice (H). The percentages indicate the proportion of each macrophage cell subtype among all macrophages at the indicated time points. The corresponding t-SNE plots show the distribution of macrophage cell subtypes at each time point. Unless otherwise indicated, these panels are descriptive and no formal statistical comparison is implied. (I) Representative multiplex immunofluorescence (mIF) images showing Trem2^+^ macrophages in Yap-Akt-induced ICC tumors and normal liver. Macrophages were identified by F4/80 staining, and Trem2 expression is shown in the corresponding channel. (J) Heatmap depicting similarity of macrophage subtypes across human, Yap-Akt, and Kras-P19 induced ICC models. This similarity was assessed by converting mouse genes to human orthologs and simulating the data using an anchor-based integration algorithm. For more detailed methodological descriptions, please refer to Materials and Methods. Abbreviations: ICC, intrahepatic cholangiocarcinoma; scRNA-seq, single-cell RNA sequencing; t-SNE, t-distributed stochastic neighbor embedding; TPM, transcripts per million; mIF, multiplex immunofluorescence.

To verify that Trem2 was preferentially expressed in macrophages of tumor tissues, we performed mIF staining on Yap-Akt-induced ICC mice, revealing that most macrophages in tumor tissues expressed Trem2, whereas Trem2 expression was scarce in normal liver tissues (Fig. 2I and Fig. S3E). A similar phenomenon was observed in the Kras-P19-induced ICC mouse model (Fig. S3F and G). Finally, to assess the transcriptional conservation of macrophage subtypes between humans and mice, we performed similarity analysis using an anchor-based algorithm. TREM2^+^ macrophages showed high similarity across both mouse models. Similarly, TIMD4^+^ macrophages showed high transcriptional similarity between human ICC samples and the Kras-P19-induced ICC mouse model, and MKI67^+^ macrophages demonstrated a high similarity between humans and mice with Yap-Akt-induced ICC. TCR^+^ macrophages exhibited a moderate similarity with Il2rb^+^ macrophages in both mouse models of ICC (Fig. 2J). Additionally, comparative analysis showed that Trem2^+^, Timd4^+^, and Plac8^+^ macrophage subtypes were highly similar between the 2 mouse models (Fig. S3H), although Plac8^+^ macrophages were not identified in human samples.

These findings identified 4 macrophage subtypes with conserved transcriptional profiles across humans and mice: Trem2^+^, Timd4^+^, TCR^+^, and Mki67^+^ macrophages. This cross-species conservation supports the use of mouse models to investigate the functional roles of corresponding macrophage subtypes in ICC.

### Profiles of macrophages during ICC development

The TME changes dynamically during tumor progression. We obtained scRNA-seq data from macrophages isolated from healthy human livers, reported by Ramachandran et al. [17] (hereafter referred to as healthy macrophages; GSE136103, Table S6), and corrected for batch effects associated with differences in sequencing platforms. In the UMAP plot, healthy macrophages clustered closely with AIF1^+^ and TIMD4^+^ macrophages and showed partial overlap with TREM2^+^ macrophages and other cell subtypes (Fig. 3A). These findings suggest that AIF1^+^ and TIMD4^+^ macrophages preserve much of the transcriptional profile of normal liver macrophages.

**Fig. 3.**
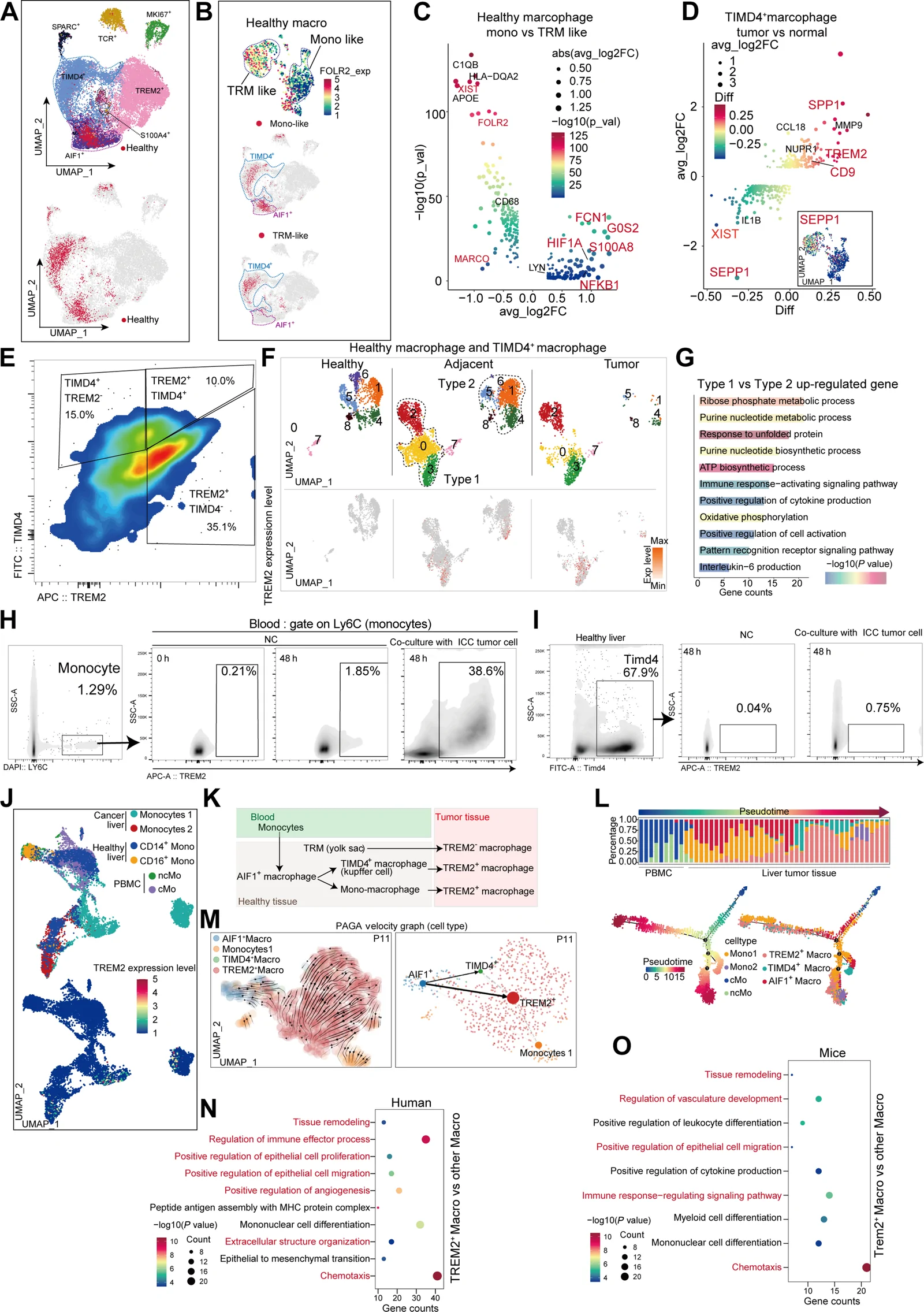
Transcriptional states and inferred developmental trajectories of macrophages in ICC. (A) UMAP visualization integrating macrophages from healthy human liver tissues with macrophages from ICC tumor and adjacent tissues. Healthy liver macrophages were obtained from the scRNA-seq dataset GSE136103 and mapped onto the integrated macrophage reference generated from ICC tumor-associated and adjacent liver tissues. The bottom panel highlights healthy liver macrophages within the integrated UMAP space. (B) UMAP plot showed 2 types of macrophage populations in normal liver tissue and their overlapping regions with macrophages from tumor-related backgrounds, including ICC tumor and adjacent nontumor tissues. (C) Volcano plot showed that differentially expressed genes (DEGs) between the 2 subtypes revealed 2 distinct states of macrophages in the healthy liver. Point size represents the absolute average log2 fold change, and color represents statistical significance, shown as −log10 adjusted *P* value. (D) Volcano plot of DEGs between TIMD4^+^ macrophage in tumor and normal tissues. Positive average log2 fold changes indicate genes enriched in tumor-derived TIMD4^+^ macrophages, whereas negative values indicate genes enriched in normal liver TIMD4^+^ macrophages. Point size represents the absolute average log2 fold change, and color represents statistical significance, shown as −log10 adjusted *P* value. (E) Representative flow cytometry analysis of TREM2 and TIMD4 expression in macrophages isolated from ICC tumor tissues. The percentages indicate the proportions of TREM2^+^TIMD4^−^, TREM2^+^TIMD4^+^, TREM2^−^TIMD4^+^, and TREM2^−^TIMD4^−^ cells within the gated macrophage population. (F) Clustering differences and TREM2 expression in TIMD4^+^ macrophages across normal liver, adjacent, and tumor tissues. (G) GO enrichment of DEGs up-regulated in Type 1 vs. Type 2 macrophages. Bar length represents gene count, and color represents −log10 adjusted *P* value. (H and I) Flow cytometry analysis of TREM2 expression in PBMC-derived monocytes (H) and normal liver TIMD4^+^ macrophages (I) after coculture with tumor cells. (J) UMAP plot of monocyte clustering and TREM2 expression in monocytes of PBMC, healthy liver, and tumor tissues. (K) Schematic of monocyte migration from PBMC to tumor mass. (L) Monocle2 pseudotime trajectory of monocyte-to-macrophage differentiation. The top panel shows changes in cell-type composition along pseudotime, and the bottom panel shows the inferred trajectory from PBMC/monocyte-associated populations toward macrophage states in liver tumor tissues. Pseudotime is an inferred transcriptional ordering and should not be interpreted as direct evidence of lineage progression. (M) PAGA and RNA velocity analysis of Monocytes 1, AIF1^+^, TIMD4^+^, and TREM2^+^ macrophage differentiation. (N and O) GO enrichment of TREM2^+^ macrophage-associated genes in humans (N) and mice (O). Bar length represents gene count, dot size represents gene count or enrichment-associated gene number, and color represents −log10 adjusted *P* value. Abbreviations: ICC, intrahepatic cholangiocarcinoma; TAM, tumor-associated macrophage; PBMC, peripheral blood mononuclear cell; UMAP, Uniform Manifold Approximation and Projection; DEG, differentially expressed gene; GO, Gene Ontology; PAGA, partition-based graph abstraction; RNA velocity, RNA-velocity analysis.

Analysis of M1/M2 polarization scores (Table S3) showed that AIF1^+^ macrophages occupied an intermediate, unpolarized state, whereas TREM2^+^ and TIMD4^+^ macrophages showed no significant polarization bias (Fig. S4A). Interestingly, a previous study reported that macrophages in the normal liver, primarily Kupffer cells, typically exhibit an M2-like phenotype [40]. This raises the question of whether Kupffer cells undergo significant changes in ICC. This prompted us to investigate whether Kupffer cells undergo substantial changes during ICC. We therefore subclustered macrophages from healthy liver samples and identified 2 distinct populations: one with typical monocytes (high expression of G0S2, FCN1, and S100A8) and the other with tissue-resident macrophages (TRMs; high expression of MARCO and FOLR2 [41]; Fig. 3B and C). Notably, the monocyte-like healthy macrophages largely overlapped with TIMD4^+^ and AIF1^+^ macrophages in the UMAP plot, whereas the TRM-like healthy macrophage cluster largely overlapped with the TIMD4^+^ macrophage cluster (Fig. 3A and B). Hierarchical clustering confirmed that the transcriptional profiles of healthy macrophages were similar to those of the AIF1^+^ and TIMD4^+^ cell subtypes (Fig. S4B), consistent with previous reports describing the dual origin of liver macrophages from yolk-sac progenitors and monocytes [42].

Surprisingly, when comparing TIMD4^+^ macrophages in tumor and adjacent tissues, we found that tumor-associated TIMD4^+^ macrophages exhibited the features of TREM2^+^ macrophages, including high expression of TREM2, CD9, and SPP1, whereas TIMD4^+^ macrophages in normal tissues retained the features of TRM macrophages (e.g., high SEPP1 expression; Fig. 3D). Similar findings were observed in AIF1^+^ macrophages (Fig. S4C). In mice, tumor-associated Timd4^+^ macrophages also showed up-regulation of Trem2-related genes, compared to those in healthy livers (Fig. S4D), implying that TIMD4 and TREM2 may be coexpressed in a subset of TAMs. As expected, flow cytometry confirmed that approximately 10% of TAMs coexpressed TREM2 and TIMD4 in ICC tumor tissue (Fig. 3E).

Comparative analysis of TIMD4^+^ macrophages from tumor, adjacent, and healthy tissues revealed that a subset of TIMD4^+^ macrophages in adjacent tissues (type 1) exhibited significant changes, including high TREM2 expression, and another subset (type 2) retained a transcriptional profile similar to that of healthy macrophages (Fig. 3F). Type 1 macrophages enhanced energy metabolism, stress responses, oxidative phosphorylation, and pattern-recognition receptor activation (Fig. 3G). Additionally, TIMD4^+^ macrophages in tumor adjacent tissues expressed higher level of FCN1, compared to healthy macrophages (Fig. S4E), suggesting that, in the tumor context, monocytes enter the liver and partially differentiate into Kupffer-like cells while up-regulating TREM2.

To validate this result, we examined Timd4^+^ macrophages from tumor tissue during ICC progression in mice. In the advanced stage (8 Weeks), nearly all Timd4^+^ macrophages expressed Fcn1 and Trem2 (Fig. S4F), indicating that Timd4^+^ macrophages in the late stage are primarily monocyte-derived and accompanied with Trem2 up-regulation. In vitro cell-coculture experiments showed that Trem2 expression was significantly up-regulated in peripheral blood monocytes when cocultured with ICC tumor cells (Fig. 3H), but Timd4^+^ macrophages from healthy livers, collected after 6 doses of anti-Csf1r treatment to deplete Kupffer cells and minimize the contribution of monocyte-derived cells, showed minimal changes in Trem2 expression (Fig. 3I and Fig. S4G). In addition, Trem2 was overexpressed in monocytes from tumor tissues, but not in normal liver tissues and peripheral blood (Fig. 3J). These findings suggest that TREM2 is a hallmark of monocyte-derived TAMs (mono-TAMs) in ICC.

Based on these observations, we proposed a developmental trajectory of macrophages in ICC: our analyses suggested that monocytes may enter the liver and acquire an AIF1^+^ macrophage-like state, with some acquiring TRM characteristics to replenish Kupffer cells and others retaining their original features; meanwhile, TREM2 expression is up-regulated in both Kupffer-like and monocyte-derived macrophages (Fig. 3K). This hypothesis was further supported by the observation that TF activity remained relatively stable in healthy and AIF1^+^ macrophages, but markedly in TIMD4^+^ and TREM2^+^ cell subtypes (Fig. S4H). Velocity, PAGA, and Monocle2 algorithms were employed for computational prediction, suggesting that AIF1^+^ macrophages may transition into TIMD4^+^ and TREM2^+^ cell subtypes (Fig. 3L and M).

To investigate whether TREM2^+^ macrophages exhibit functional similarities between humans and mice, we performed GO enrichment analysis on the signature genes (defined as genes up-regulated relative to other macrophage cell subtypes) of TREM2^+^ macrophages. The results indicated that TREM2^+^ macrophages in both humans and mice exhibited similar roles in ICC development, including tissue remodeling, immune regulation, promotion of epithelial cell proliferation and migration, angiogenesis, and chemotaxis (Fig. 3N and O). Additionally, TREM2^+^ macrophages were major contributors to the generation of chemokines (such as CXCL10 [43] and CXCL9 [44]), highlighting their role in shaping the TIME (Fig. S4I), particularly through TGF-β production and activation (Fig. S4J).

In summary, as indicated by the lineage-tracing studies of Kupffer cells by Schulz et al. [45] and Christofides et al. [46], we proposed a developmental trajectory of TAMs in ICC. TREM2 serves as a characteristic marker of monocyte-derived and TREM2^+^ macrophages. TIMD4^+^ macrophages exhibit significant changes in adjacent tissues, despite the absence of direct tumor cell infiltration, suggesting the need to redefine the state of TAMs.

### TREM2^+^ macrophages as a contributor to the poor prognosis of ICC

The Scissor algorithm was used to identify cell populations (Scissor^+^ cells) associated with poor prognosis in patients with ICC. This analysis identified 517 cells markedly associated with poor prognosis in macrophages (Scissor^+^ subset, Fig. 4A), and these cells were predominantly distributed within the TREM2^+^ macrophage cluster, indicating that TREM2^+^ macrophages may be a key determinant of unfavorable clinical outcomes. Furthermore, TREM2 was preferentially expressed by macrophages in both human and mouse samples (Fig. S5A and B). This specificity supports the use of TREM2 as a marker for identifying TREM2^+^ macrophages and highlighting the therapeutic potential of targeting TREM2^+^ macrophages. Survival analysis was performed in 2 independent ICC cohorts (FuDan-ICC cohort and EHBH-ICC cohort), confirming that higher TREM2 expression was significantly associated with shorter overall survival (Fig. 4B and Fig. S5C). Consistent results were observed in a pan-cancer cohort (Xena pan-cancer cohort; Fig. 4B and Table S6). Patients with higher TREM2 levels had higher rates of intrahepatic metastasis, vascular invasion, and lymph node metastasis, as well as more advanced clinical stages (Fig. 4C and D). IHC staining of tumor tissues from 39 ICC patients further demonstrated a progressive increase in TREM2 expression with advancing clinical stage and the presence of lymph node metastasis (Fig. 4E and F).

**Fig. 4.**
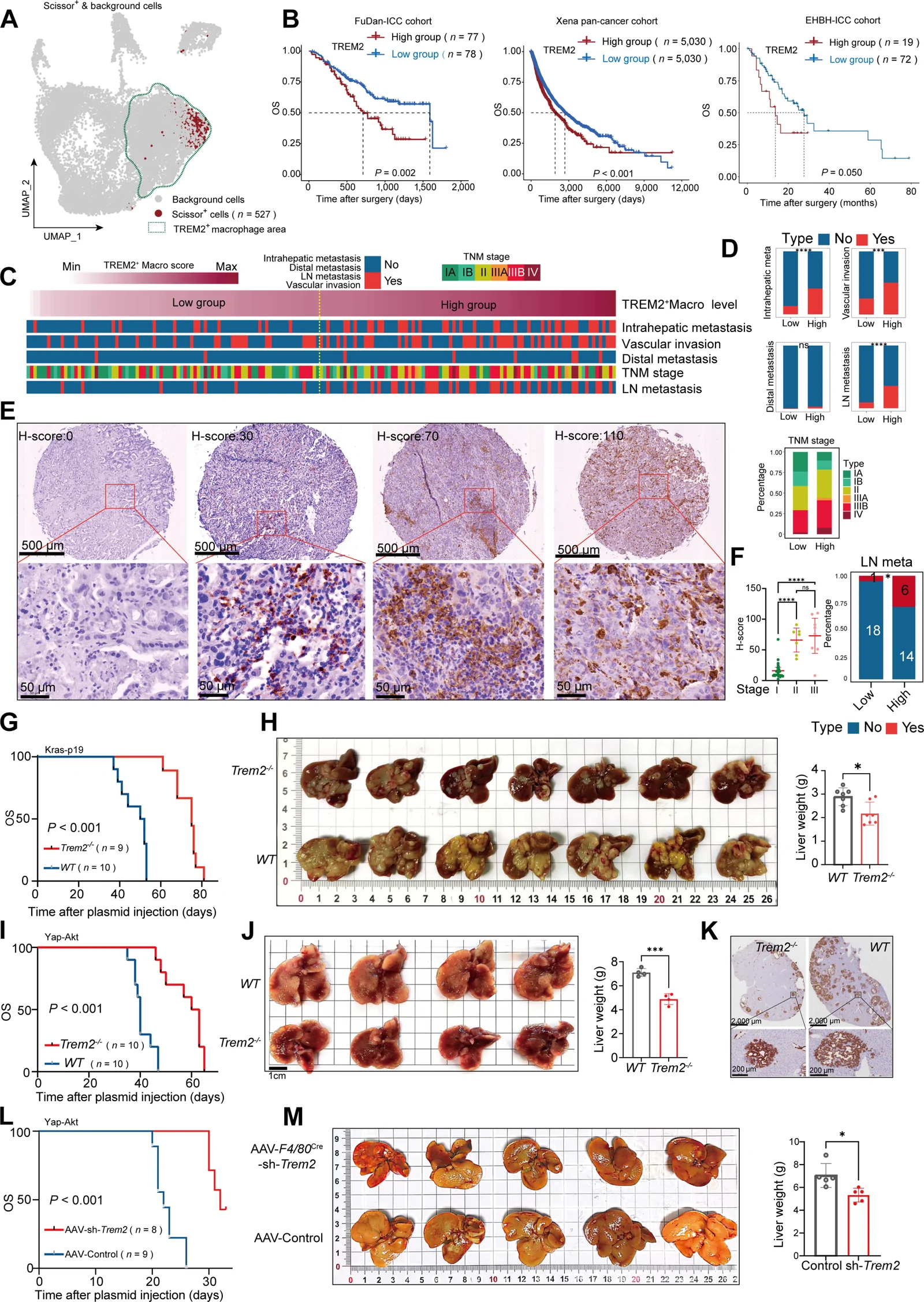
TREM2^+^ macrophages are associated with adverse clinical outcomes and promote ICC progression. (A) UMAP visualization of Scissor^+^ and background cells identified by integrating single-cell transcriptomic data with patient-level survival information. Scissor^+^ cells represent individual cells whose transcriptional profiles are associated with the adverse clinical outcome of ICC patients; background cells are shown for reference. The TREM2^+^ macrophage compartment is outlined in green, and Scissor^+^ cells are highlighted in red. (B) Kaplan–Meier overall-survival analyses according to TREM2^+^ macrophage-associated scores in the FuDan-ICC cohort, the Xena pan-cancer cohort, and the EHBH-ICC cohort. Patients were stratified into TREM2-high and TREM2-low groups using the prespecified cutoff for each cohort. Survival distributions were compared using the log-rank test. The numbers of patients in each group are indicated in the plots. (C and D) Clinicopathological characteristics of TREM2-high and TREM2-low groups in the FuDan-ICC cohort. The incidence rates of vascular invasion, intrahepatic metastasis, distal metastasis, and LN metastasis in ICC patients (C). Statistical comparison of clinical phenotype counts between the high- and low-TREM2^+^ macrophage groups (D) (^***^P < 0.001; ^****^P < 0.0001; ns, not significant; χ^2^ test). (E) IHC H-score of TREM2 association with clinicopathological features in ICC tumor tissues (Table S1; ^****^P < 0.0001; ns, not significant; one-way ANOVA followed by Dunnett’s tests; LN metastasis, ^*^P < 0.05; χ^2^ test). (F) Statistical differences in stage and lymph node metastasis between the high- and low-TREM2 H-score groups (stage I, *n* = 24; stage II, *n* = 7; stage III, *n* = 8; ^*^P < 0.05; Kruskal–Wallis test followed by Dunn’s multiple-comparisons test). (G and H) Survival and tumor burden in Kras-P19-induced ICC mice with or without *Trem2* genetic deletion (G). Kaplan–Meier survival analysis (H). Representative liver images and quantification of liver weight at the experimental endpoint. Survival curves were compared using the log-rank test, and liver weights were compared using a 2-tailed Mann–Whitney *U* test (^*^P < 0.05; ^***^P < 0.001). The number of mice in each group is indicated in the plots. (I and J) Survival and tumor burden in Yap-Akt-induced ICC mice with or without *Trem2* genetic deletion (I). Kaplan–Meier survival analysis (J). Representative liver images and quantification of liver weight at the experimental endpoint. Survival curves were compared using the log-rank test, and liver weights were compared using a 2-tailed Mann–Whitney *U* test (^***^P < 0.001). The number of mice in each group is indicated in the plots. (K) Immunohistochemical staining showed the expression levels of CK19 in the *Trem2^−/−^* and WT groups tumor tissues. (L and M) Survival and tumor burden in Yap-Akt-induced ICC mice treated with AAV-*F4/80*^Cre^-sh-*Trem2* or the corresponding AAV control (L). Kaplan–Meier survival analysis (M). Representative liver images and quantification of liver weight at the experimental endpoint. Survival curves were compared using the log-rank test, and liver weights were compared using a 2-tailed Mann–Whitney *U* test (^*^P < 0.05). The number of mice in each group is indicated in the plots. Abbreviations: ICC, intrahepatic cholangiocarcinoma; UMAP, Uniform Manifold Approximation and Projection; OS, overall survival; H-score, histological score; TNM, tumor-node-metastasis; LN, lymph node; WT, wild type; AAV, adeno-associated virus; mIF, multiplex immunofluorescence.

To investigate the tumor-promoting role of TREM2 in ICC, we generated *Trem2* knockout (*Trem2^−/−^*) C57BL/6J mice. Compared to wild-type (WT) mice, *Trem2^−/−^* mice exhibited a significantly slower tumor progression, a phenotype conserved in both Kras-P19- and Yap-Akt-induced ICC (Fig. 4G to K and Fig. S5D). Furthermore, we also observed that the expression levels of genes associated with TREM2^+^ macrophages gradually increased with tumor progression in Yap-Akt-induced ICC mice (Fig. S5E). *Trem2^−/−^* mice also demonstrated a prolonged survival and reduced tumor burden compared with the WT group (Fig. 4G to K). To assess the macrophage-specific contribution of Trem2, we specifically silenced *Trem2* in macrophages using *F4/80^Cre^*-sh-*Trem2* AAV8 adenovirus. Macrophage-specific *Trem2* knockdown significantly delayed tumor progression compared to control mice (Fig. 4L and M and Fig. S5F), confirming the myeloid-specific contribution of Trem2 to ICC pathogenesis.

In summary, elevated TREM2^+^ macrophage levels were associated with adverse outcomes in ICC. Genetic or myeloid-specific inhibition of TREM2 markedly suppresses tumor progression in mouse models, aligning with prior evidence that TREM2^+^ macrophages exhibit pro-tumorigenic functions [14–16,47]. These findings underscore the therapeutic potential of targeting TREM2^+^ macrophages in ICC.

### Effects of Trem2 knockout on the myeloid TME in ICC

Based on human ICC samples stratified by TREM2 expression levels, the bulk RNA-seq analysis further revealed that high-TREM2 tumor tissues presented activations of pathways related to immune regulation, chemotaxis, epithelial proliferation/migration, macrophage differentiation, and mesenchymal differentiation (Fig. S6A). At the same time, to investigate how *Trem2* knockout delays ICC progression, we performed scRNA-seq on tumor tissues collected from *Trem2^−/−^* and WT mice 8 weeks after injection of the Kras-P19 plasmid, as well as on normal liver tissues from control mice. As anticipated, *Trem2* deficiency reduced the abundance of myeloid cells in *Trem2^−/−^* mice compared with WT mice (Fig. 5A and Fig. S6B to D). Surprisingly, we also observed marked decreases in the populations of fibroblasts and endothelial cells, alongside a significant increase in the NK/T cell ratio (Fig. 5A and Fig. S6B to D). Among immune-cell subsets, the proportion of NK/T cells was also increased among CD45^+^ cells (Fig. 5B), and these findings are discussed in the following section. Next, in the subclusters of myeloid populations (Fig. 5C and Fig. S6E), *Trem2* knockout significantly reduced Trem2 expression in macrophages (Fig. S6F). Flow cytometry confirmed the decreased expression of monocyte marker (Ly6c), myeloid marker (Cd11b), and macrophage marker (*F4/80*) in *Trem2^−/−^* ICC mice (Fig. 5D and E and Fig. S6G), suggesting that TREM2 may be involved in the transition from monocytes to macrophages. Strikingly, Kupffer cells exhibited the most prominent reduction in *Trem2^−/−^* ICC mice (Fig. 5F and G). Given the high *Trem2* expression in Kupffer cells (Fig. 5H and Fig. S6H), it suggests that most of the tumor-associated Kupffer cells originate from monocytes during advanced-stage ICC, aligning with our mechanistic conclusions.

**Fig. 5.**
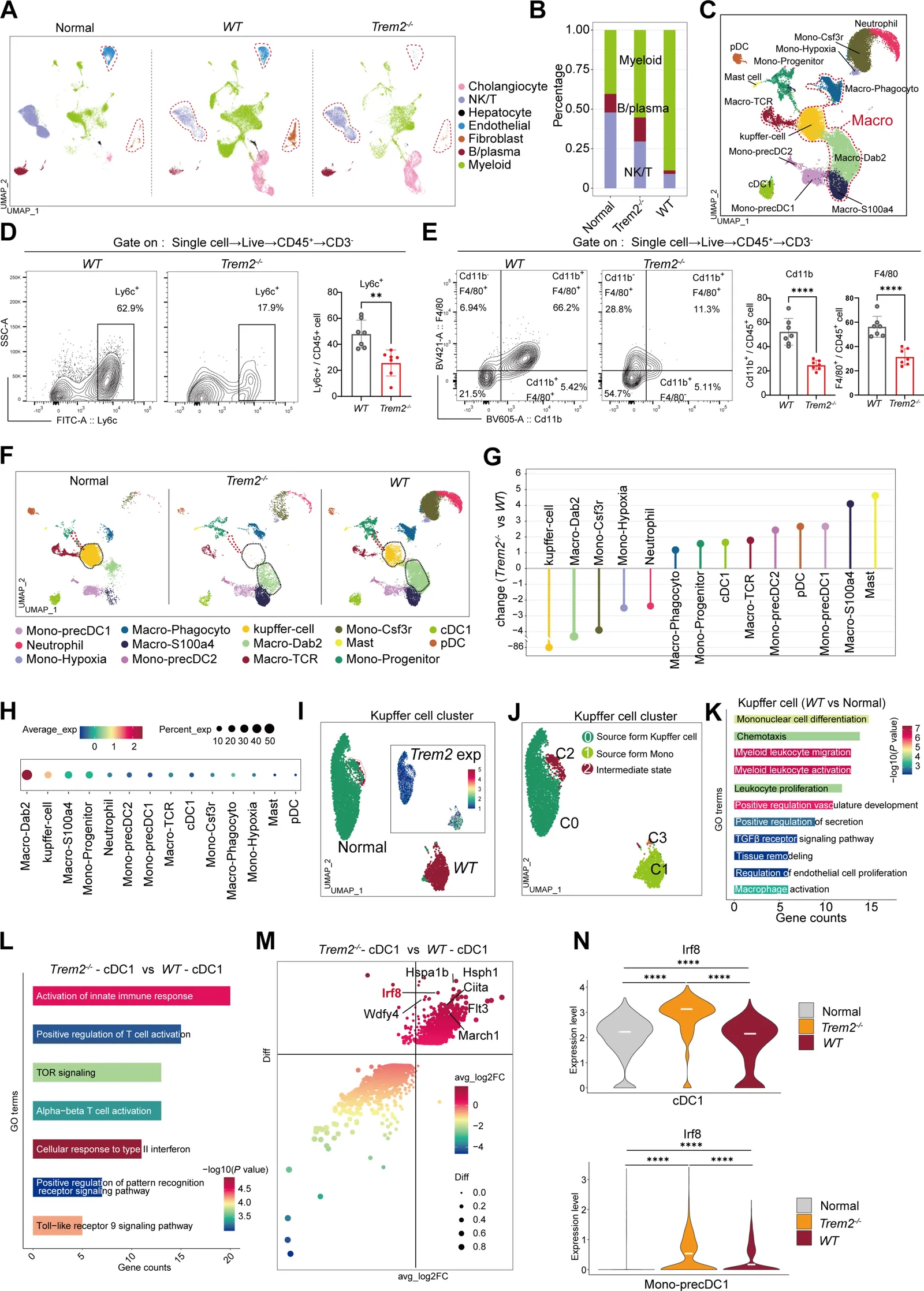
Remodeling of *Trem2* knockout on the TME in ICC. (A) UMAP visualization of scRNA-seq profiles from normal liver, wild-type (WT) Kras-P19-induced ICC tumors, and *Trem2^−/−^* Kras-P19-induced ICC tumors. Major cell populations are annotated according to their marker-gene expression profiles. (B) Statistical analysis based on the proportion of cell types within each group. Differential leukocyte composition across normal liver, *Trem2^−/−^*, and WT ICC mice liver. The bar chart illustrates the proportion of each cell type within the total leukocyte population in the corresponding tissues. (C) Myeloid cell distributions in the normal, WT, and *Trem2^−/−^* groups were analyzed by scRNA-seq and visualized using UMAP plots. (D and E) Flow cytometry analysis of myeloid cell markers in *Trem2^−/−^* vs. WT ICC mice tumors (monocyte marker, Ly6C; monocyte/macrophage marker, CD11b; and macrophage marker, F4/80; ^**^P < 0.01; ^****^P < 0.0001; 2-tailed Mann–Whitney *U* test). (F) UMAP visualization of the myeloid-cell compartment showing the distribution of annotated myeloid subclusters in normal liver, *Trem2^−/−^* ICC tumors, and WT ICC tumors. (G) Differential abundance analysis of myeloid cell subtype between *Trem2^−/−^* and WT ICC tumors based on scRNA-seq data. The swimming plot shows the relative changes in the abundance of each myeloid cell subtypes in *Trem2^−/−^* tumors compared with WT tumors. The statistical model and multiple-testing correction are described in Materials and Methods. (H) Dot plot showed *Trem2* expression across myeloid cell subtypes based on scRNA-seq. Dot size indicates the percentage of cells expressing *Trem2*, and color intensity represents the average expression level. (I and J) scRNA-seq analysis of *Trem2* expression and tissue distribution in Kupffer cells. UMAP visualization of the Kupffer cell cluster showed *Trem2* expression and the distribution of Kupffer cells derived from normal liver, WT ICC tumors, and *Trem2^−/−^* ICC tumors (I). Kupffer cells were further divided into 4 subtypes, C0 to C3. C0 represents Kupffer cells mainly derived from normal liver, C1 represents Kupffer cells mainly associated with WT tumors, C2 represents intermediate-state Kupffer cells, and C3 represents Kupffer cells enriched in the *Trem2^−/−^* tumor background (J). (K) GO enrichment analysis of genes up-regulated in tumor-associated Kupffer cells compared with normal Kupffer cells, based on scRNA-seq-derived differential gene expression analysis. (L and M) GO enrichment (L) and DEGs (M) of cDC1 cells detected by scRNA-seq analysis in *Trem2^−/−^* versus WT tumors. The genes for GO enrichment were derived from the up-regulated DEGs in cDC1 cells from *Trem2^−/−^* mice compared with WT mice. (N) Irf8 expression levels detected by scRNA-seq DEGs analysis in cDC1 and Mono-preDC1 cells from *Trem2^−/−^* and WT. Following *Trem2* knockout (^****^P < 0.0001; Dunn’s multiple-comparisons test). Abbreviations: ICC, intrahepatic cholangiocarcinoma; WT, wild type; UMAP, Uniform Manifold Approximation and Projection; scRNA-seq, single-cell RNA sequencing; GO, Gene Ontology; DEG, differentially expressed gene; cDC1, conventional type 1 dendritic cell; Mono-preDC1, monocyte/pre-cDC1 population; TME, tumor microenvironment.

Kupffer cells were further subclustered into cluster 0 (C0), which was enriched in normal liver; cluster 1 (C1), which predominated in tumor tissues from WT mice; and cluster 2 (C2), which represented an intermediate state (Fig. 5I and J). Because the C3 subcluster represented only a very small fraction of cells, it was considered outside the scope of this study and was not discussed further. In WT mice, Kupffer cells in cluster 1 (C1) expressed high levels of monocyte- and TREM2^+^ macrophage-associated genes (Fig. S6I and J). Comparative analysis further revealed that monocytic and TREM2^+^ macrophage signature genes, including S100a8, S100a9, Trem2, Spp1, Cd9, and G0s2, were up-regulated in WT tumor-associated clusters compared with normal clusters (Fig. S6K). Functional annotation of WT tumor-associated Kupffer cells demonstrated the enrichment of pathways related to myeloid cell action, angiogenesis regulation, TGF-β signaling, tissue remodeling, and endothelial proliferation (Fig. 5K), underscoring the pivotal role of Trem2 in reprogramming monocyte-derived macrophages, including their differentiation toward Kupffer-like phenotypes.

In addition, the proportion of DCs increased in the tumor tissues of *Trem2^−/−^* mice (Fig. 5G). Specifically, cDC1 cells showed increased activity of pathways related to T cell activation, TOR signaling, and pattern recognition receptor activity following *Trem2* knockout (Fig. 5L), accompanied by increased Irf8 expression (Fig. 5M and N). Previous findings have shown that Irf8 is involved in cDC1 differentiation and maturation [48], which are essential for effective antitumor immunity. These findings suggest that *Trem2* ablation may alleviate macrophage-mediated suppression of cDC1 activity, thus potentiating the antitumor responses of CD8^+^ T cells.

Overall, *Trem2* knockout reduced the abundance of monocytes and macrophages in the mouse ICC model and revealed that late-stage Kupffer cells were predominantly replenished by monocyte-derived precursors with elevated TREM2 expression. Concurrently, the NK/T cell ratio and cDC1 proportion increased, while fibroblasts and endothelial cells declined. These microenvironmental alterations mechanistically elucidate the role of TREM2 in promoting ICC progression.

### TREM2^+^ macrophages promote ICC invasion through the PLAU–PLAUR axis

We found that TREM2^+^ macrophages not only modulated the TIME, but also directly enhanced tumor aggressiveness, particularly in patients with high TREM2 expression, who were more likely to develop intrahepatic and lymph node metastases (Fig. 4C). To investigate the direct role of TREM2^+^ macrophages in propelling ICC invasion, we compared tumor cells from *Trem2^−/−^* and WT mice. The tumor cells from WT mice showed up-regulated expression of Spp1, Sparc, Vim, and S100a4 (Fig. S7A), all of which are genes associated with enhanced migration, invasion, and epithelial–mesenchymal transition ([49,50]). This finding was corroborated by GO enrichment analysis (Fig. S7B).

CellPhoneDB analysis of macrophages–malignant cells interactions revealed that TREM2^+^ macrophages showed the highest interaction scores with malignant cells (Fig. S7C). The urokinase-type plasminogen activator–urokinase-type plasminogen activator receptor (PLAU–PLAUR) ligand–receptor pair was specifically enriched in TREM2^+^ macrophages, including Scissor^+^ macrophages, in tumor tissues (Fig. S7D). PLAUR was expressed in both malignant cells and myeloid cells (Fig. S7E and F), and PLAU was predominantly expressed by myeloid cells. Within the macrophage compartment, PLAU expression was mainly observed in TREM2^+^ and MKI67^+^ macrophage subtypes (Fig. S7E and G). IHC staining confirmed that PLAUR expression was higher in human tumor tissues than in paired normal tissues (Fig. S7H). mIF staining validated the coexistence of PLAU^+^ TREM2^+^ macrophages and PLAUR^+^ malignant cell (Fig. S7I and J). Spatial transcriptomics further highlighted the enrichment of PLAU^+^ TREM2^+^ macrophages at the tumor border (Fig. S7K). In mice, PLAU and PLAUR were markedly up-regulated in tumor tissues, particularly in Kras-P19-induced ICC tumors (GSE171443 cohort, Table S4 and Fig. S7L). Clinically, higher PLAU and PLAUR expression levels were associated with poor prognosis, advanced clinical stage, and lymph node metastasis in ICC patients (Fig. S7M to O).

To determine whether TREM2^+^ macrophages promote ICC invasion via the PLAU–PLAUR axis, we measured PLAU protein levels by ELISA, finding that its level was significantly higher in TREM2^+^ versus TREM2^−^ macrophages (Fig. S8A). Subsequently, we detected PLAUR expression levels in human ICC cell lines and the normal human bile duct epithelial cell line HiBEpiC by Western blotting. Because HCCC-9810 and HuCCT1 cells showed the highest PLAUR expression, they were selected for subsequent functional experiments (Fig. S8B). Coculture with human TREM2^+^ macrophages isolated from ICC patients markedly increased the migration and invasion of HuCCT1 cells compared with the corresponding control condition, while this effect was significantly attenuated by the PLAU–PLAUR inhibitor IPR-803. Notably, IPR-803 alone did not significantly affect the migration or invasion of ICC cells compared with the NC group (Fig. S8C and D). Furthermore, by adding recombinant PLAU protein, with or without the IPR-803, to the cell culture medium, we observed that PLAU promoted the invasion and migration of tumor cells, an effect that could be reversed by IPR-803 (Fig. S8D and E).

To further validate the roles of PLAUR in ICC, we selectively silenced *Plaur* in malignant cells using an AAV8 vector administered by tail vein injection to 3-week-old C57BL/6J mice. The model was induced by Kras-P19 plasmid injection, and the mice were sacrificed 8 weeks after injection. Compared with the control mice, AAV8-mediated silencing effectively reduced Plaur expression in malignant cells (Fig. S8F), while simultaneously reducing tumor burden (Fig. S8G) and the incidence of lung metastasis (Fig. S8H). Correspondingly, silencing *Plaur* in tumor cells resulted in a modest extension of survival in mice (Fig. S8I). Additionally, in vivo administration of IPR-803 was able to slow tumor progression and reduce the incidence of lung metastasis (Fig. S8J and K). These results demonstrated that TREM2^+^ macrophages accelerated ICC progression and metastatic dissemination by secreting PLAU to activate PLAUR on tumor cells.

### TREM2^+^ macrophages promote POSTN^+^ CAF formation at the tumor–peritumor interface to restrict CD8^+^ T cell infiltration in ICC

*Trem2* knockout markedly reduced fibroblasts in the ICC mice (Fig. S9A). ICC is a desmoplastic tumor rich in fibroblasts, and the role of cancer-associated fibroblasts (CAFs) in tumor has been well documented, such as in mechanisms like IL-6 secretion by inflammatory CAF (iCAF; [51]) and hyaluronic acid production by myofibroblastic CAF (myCAF; [52]). Given these, we hypothesized that CAFs might mediate TREM2-driven ICC progression. DEGs and functional enrichment analyses were performed on fibroblasts from *Trem2^−/−^* and WT mice, demonstrating down-regulation of Postn, Vegfa, and activation of pathways linked to TGF-β signaling, stromal development, collagen deposition, and cell migration in *Trem2^−/−^* mice (Fig. 6A and B), suggesting that TREM2^+^ macrophages drive fibroblast differentiation into collagen-producing, extracellular matrix-remodeling subtypes.

**Fig. 6.**
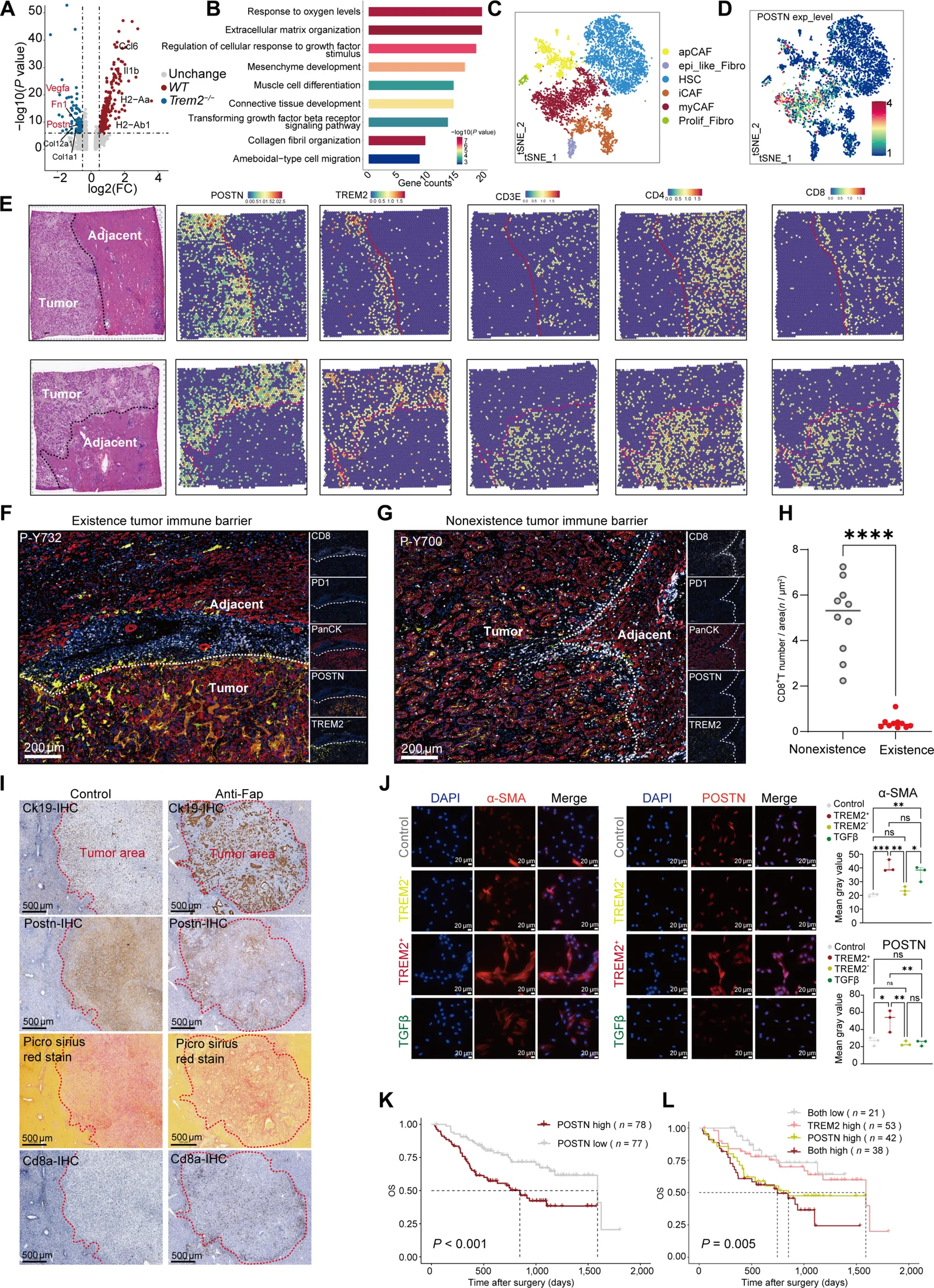
TREM2^+^ macrophages promote POSTN^+^ CAF formation at the tumor boundary to establish an immune barrier restricting CD8^+^ T cell infiltration in ICC. (A and B) DEGs analysis (A) and GO enrichment (B) of up-regulated genes in fibroblasts from *Trem2^−/−^* versus WT tumors (Kras-p19-induced ICC mice). (C and D) UMAP plot of human fibroblast cell subtypes (C) and POSTN expression levels (D). (E) Spatial transcriptomics shows expression level and distribution of genes in ICC samples. The dotted line demarcates the boundary between the tumor and peritumoral tissue. (F to H) mIF staining showing spatial distribution (F and G) and quantitative analysis (H) of CD8^+^ T cells in the presence or absence of an immune barrier (^****^P < 0.0001; Mann–Whitney *U* test). (I) IHC was performed to evaluate the expression levels of Ck19, Postn, and Cd8a, and SR was performed to evaluate collagen abundance, between the anti-Fap treatment group and the control group. For detailed procedures regarding the preparation of the anti-Fap therapeutic agent, please refer to Materials and Methods. (J) α-SMA and POSTN expression detected by mIF staining in LX-2 cocultured with TREM2^+^ macrophages or TREM2^−^ macrophages or treated with TGF-β (^*^P < 0.05; ^**^P < 0.01; ^***^P < 0.001; ns, not significant; one-way ANOVA with Dunnett’s test). (K and L) Survival (Kaplan–Meier) analysis of POSTN (K) and TREM2-POSTN coexpression (L) in the FuDan-ICC cohort. Abbreviations: ICC, intrahepatic cholangiocarcinoma; CAF, cancer-associated fibroblast; DEGs, differentially expressed genes; UMAP, Uniform Manifold Approximation and Projection; GO, Gene Ontology; WT, wild type; apCAF, antigen-presenting cancer-associated fibroblast; HSC, hepatic stellate cell; iCAF, inflammatory cancer-associated fibroblast; myCAF, myofibroblastic cancer-associated fibroblast; Prolif_Fibro, proliferating fibroblast; IHC, immunohistochemistry; PanCK, pan-cytokeratin; FAP, fibroblast activation protein; OS, overall survival.

Furthermore, a total of 6,544 fibroblasts from human samples were identified and classified into 6 cell subtypes (Fig. 6C and Fig. S9B), including a POSTN^+^ CAF subset (myCAF) markedly enriched in tumor tissues (Fig. 6D and Fig. S9C). POSTN^+^ CAFs were also elevated in *Yap*-, *Kras*-, and *Fbxw7*-driven ICC models (Fig. S9D), confirming their tumor-specific role. The bulk RNA-seq data failed to correlate TREM2 with POSTN^+^ CAF signatures (Fig. S9E), likely due to bulk RNA-seq analysis masking spatial heterogeneity. However, spatial transcriptomic analysis revealed that TREM2^+^ macrophages and POSTN^+^ CAFs were colocalized in the peritumoral region (Fig. 6E). This spatial organization was associated with the formation of an immune-excluding barrier at the tumor margin, where CD8^+^ T cell infiltration into the tumor core was markedly reduced. This finding is consistent with a previous report by Liu et al. [53], which showed that SPP1^+^ macrophages and CAFs formed an immune-excluding border that reduced the efficacy of anti-PD-1 therapy in hepatocellular carcinoma. In the retrospective analysis of noncirrhotic ICC margin tissues, mIF staining confirmed that tumors with TREM2^+^ macrophages and POSTN^+^ CAF barriers exhibited a significantly reduced infiltration of intratumor CD8^+^ T cells (Fig. 6F to H and Fig. S9F to I).

To validate that myCAF indeed impede the infiltration of CD8^+^ T cells into tumor tissues, we pharmacologically induced myCAF quiescence. Specifically, we found that both POSTN and FAP were specifically expressed in myCAF (Fig. S9J). Therefore, we utilized pirfenidone (an anti-fibrosis drug) encapsulated in Fap-targeting nanospheres to induce myCAF quiescence. After treatment, the hepatic tumor burden was reduced (Fig. 6I and Fig. S9K and L). Furthermore, mice showed reduced Postn expression (Fig. 6I and Fig. S9M) and collagen deposition in tumor tissues (Fig. 6I and Fig. S9N), accompanied by increased CD8^+^ T cell infiltration (Fig. 6I and Fig. S9O).

To test whether TREM2^+^ macrophages promote the activation of hepatic stellate cells (HSCs) toward a myCAF phenotype, we cocultured quiescent LX-2 HSCs with TREM2^+^ or TREM2^−^ macrophages. TREM2^+^ macrophages induced α-SMA expression and morphological activation in LX-2 cells, alongside POSTN (a hallmark of myCAF) up-regulation (Fig. 6J). Notably, TGF-β alone activated LX-2 cells but did not enhance POSTN expression, suggesting that TREM2^+^ macrophages specifically drive myCAF differentiation.

These findings suggest that TREM2^+^ macrophages at the tumor–peritumor interface promote the acquisition of a POSTN^+^ myCAF-like phenotype in HSCs. The spatial association of these cells with reduced intratumoral CD8^+^ T cell infiltration may contribute to an immunosuppressive microenvironment and ICC progression. Clinically, higher POSTN expression was associated with a poor prognosis, and patients with high expression of both TREM2 and POSTN exhibited the worst outcomes (Fig. 6K and L).

Mechanistically, the ligand–receptor analysis suggested that the EREG–EGFR and PDGFB–PDGFRB axes may contribute to TREM2^+^ macrophage-associated POSTN^+^ CAF differentiation, while POSTN^+^ CAF might reciprocally modulate TREM2^+^ macrophages via TGF-β signaling (Fig. S9P), though this feedback loop requires further validation. Collectively, these findings suggest that TREM2^+^ macrophages promote the differentiation of HSCs toward a POSTN^+^ myCAF phenotype and contribute to the formation of an immunosuppressive barrier that restricts CD8^+^ T cell infiltration and may promote ICC progression.

### TREM2^+^ macrophages promote CD8^+^ T cell exhaustion and Treg accumulation, contributing to an immunosuppressive ICC microenvironment

Genetic ablation of *Trem2* in ICC mice increased the proportion of NK/T cells (Fig. 5A and B). Given the established immunomodulatory functions of TREM2^+^ macrophages [14–16,29], we hypothesized that Trem2 deficiency might reshape the composition and functional state of lymphoid cells in the ICC TIME. We therefore profiled NK/T cell populations in tumors from *Trem2^−/−^* and WT mice.

Integrated analysis of NK/T cells from *Trem2^−/−^* and WT mice identified 5 CD8^+^ T cell subtypes, 3 CD4^+^ T cell subtypes, 2 NK cell subtypes, 1 NKT cell subtype, and 1 IL23R^+^ T cell population (Fig. 7A and Fig. S10A and B). Compared with WT tumors, *Trem2^−/−^* tumors showed higher naive and activation scores but lower exhaustion scores and TCR-signaling scores (Fig. 7B). Consistent with these findings, quantitative analysis revealed an increased proportion of naive CD8^+^ T cells and GZMA^+^ NK cells, together with reduced proportions of Tregs, CD8^+^ exhausted T cells (CD8^+^ Tex), and NKT cells after *Trem2* knockout (Fig. 7C and Fig. S10C and D).

**Fig. 7.**
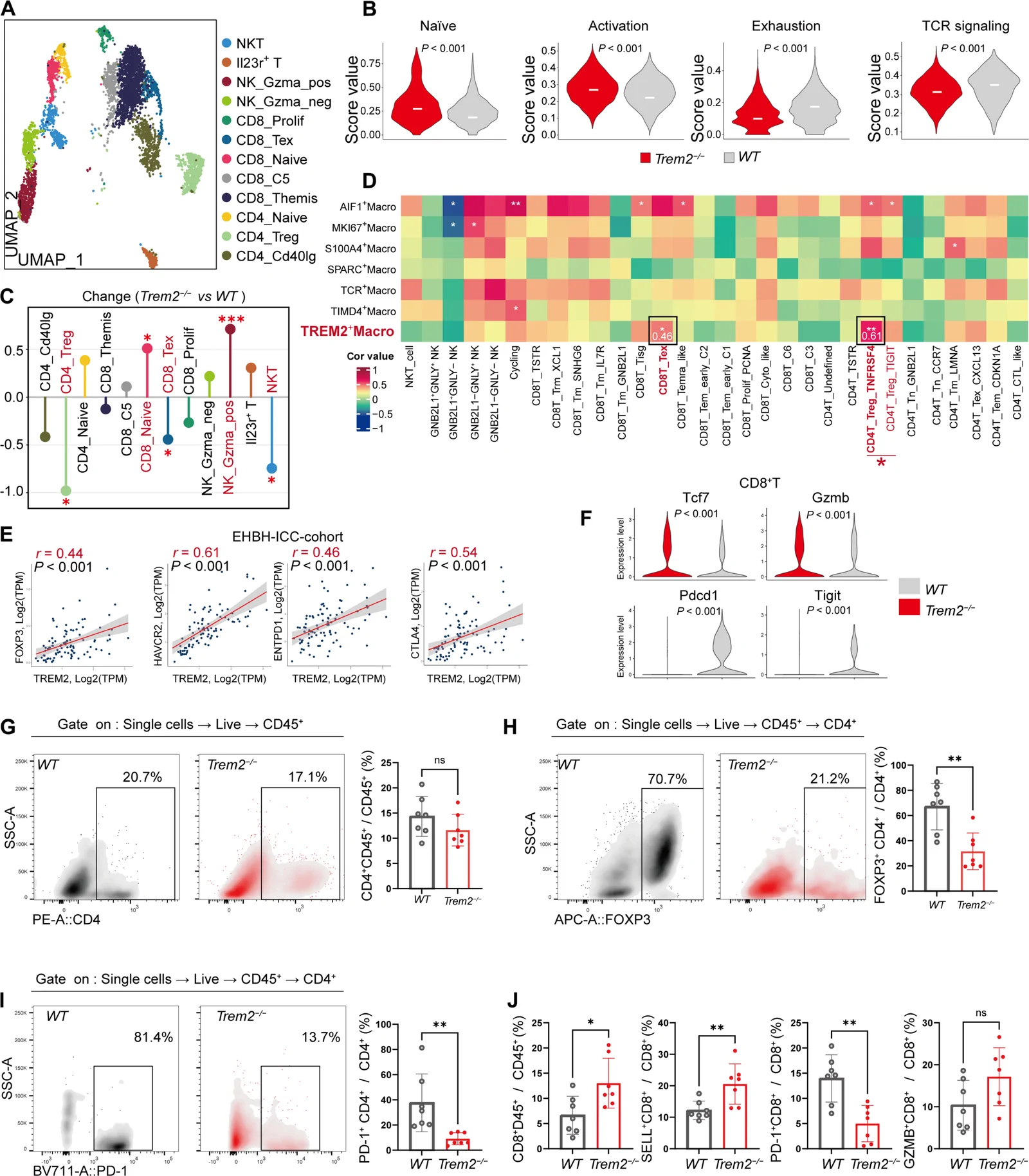
TREM2^+^ macrophages promote immunosuppressive microenvironment in ICC by regulating CD8^+^ T cell exhaustion and Treg development. (A) UMAP visualization of NK and T cell subpopulations in tumor tissues from *Trem2^WT^* and *Trem2^−/−^* ICC mice. (B) Enrichment score of T cell-related signatures in T cells (excluded NKT cell). Following *Trem2* knockout (2-tailed Mann–Whitney *U* test). (C) Ratios of NK or T cells in total leukocytes from *Trem2^−/−^* versus WT tumors (^*^P < 0.05; ^***^P < 0.001; Mann–Whitney *U* test). (D) Heatmap of Spearman correlations between macrophage subcell type and NK or T subcell type proportions in ICC samples (^*^P < 0.05, ^**^P < 0.01; multiple testing using the Benjamini–Hochberg procedure, Spearman correlations). (E) Spearman correlation of TREM2 with immunosuppressive markers (FOXP3, HAVCR2, ENTPD1, and CTLA4) in the EHBH-ICC cohort (the mRNA expression levels were TPM-transformed and subsequently log2-normalized for correlation analysis). (F) Differential expression of Tcf7, Gzmb, Pdcd1, and Tigit in CD8^+^ T cells from *Trem2^−/−^* versus WT tumors (2-tailed Mann–Whitney *U* test). (G) Flow cytometry analysis of Cd4^+^ T cell proportions in *WT* and *Trem2^−/−^* ICC mouse tumors. Cells were gated sequentially on single cells, live cells, and leukocytes (Cd45^+^ cells). Representative flow cytometry plots and quantification of Cd4^+^ cells among leukocytes are shown (ns, not significant; one-way ANOVA). (H) Flow cytometry analysis of Foxp3^+^Cd4^+^ regulatory T cells in *WT* and *Trem2^−/−^* ICC mouse tumors. Cells were gated sequentially on single cells, live cells, Cd45^+^ cells, and Cd4^+^ T cells. Representative flow cytometry plots and quantification of Foxp3^+^Cd4^+^ cells among Cd4^+^ T cells are shown (^**^P < 0.01; one-way ANOVA). (I) Flow cytometry analysis of Pd-1 expression in Cd4^+^ T cells from *WT* and *Trem2^−/−^* ICC mouse tumors. Cells were gated sequentially on single cells, live cells, Cd45^+^ cells, and Cd4^+^ T cells. Representative flow cytometry plots and quantification of Pd-1^+^Cd4^+^ cells among Cd4^+^ T cells are shown (^**^P < 0.01; one-way ANOVA). (J) Flow cytometry quantification of Cd8^+^ T cell proportions and Cd8^+^ T cell marker expression in *WT* and T*rem2^−/−^* ICC mouse tumors. The proportions of Cd8^+^ cells among Cd45^+^ cells, Sell^+^Cd8^+^ cells among Cd8^+^ T cells, Pd-1^+^Cd8^+^ cells among Cd8^+^ T cells, and Gzmb^+^Cd8^+^ cells among Cd8^+^ T cells are shown (^**^P < 0.05; ^**^P < 0.01; ns, not significant; one-way ANOVA). Abbreviations: ICC, intrahepatic cholangiocarcinoma; UMAP, Uniform Manifold Approximation and Projection; WT, wild type; TCR, T cell receptor; NK, natural killer; NKT, natural killer T; Treg, regulatory T cell; TPM, transcripts per million; PD-1, programmed cell death protein 1; GZMB, granzyme B.

These results suggested that TREM2 deficiency is associated with a less exhausted CD8^+^ T cell state and reduced Treg abundance. To examine whether similar patterns were present in human ICC, we performed high-resolution clustering of NK and T cells from human ICC samples, identifying 14 CD8^+^ T cell subsets, 10 CD4^+^ T cell subsets, 4 NK cell subsets, 1 NKT cell subset, and circulating T cells (Fig. S11A to H). Consistent with previous studies [7,54,55], tumor tissues were enriched in CXCL13^hi^CD4^+^ T cells, Tregs, and CD8^+^ Tex (Fig. S11I).

Correlation-network analysis showed moderate positive associations between TREM2^+^ macrophages and CD8^+^ Tex (*r* = 0.46) and between TREM2^+^ macrophages and TNFRSF4^hi^ Treg (*r* = 0.61), whereas the association with TIGIT^hi^ Tregs did not reach statistical significance (Fig. 7D). In an independent cohort, TREM2 expression was positively correlated with FOXP3, HAVCR2, ENTPD1, and CTLA4 expression (Fig. 7E and Fig. S10E), further supporting an association between TREM2 expression and immunosuppressive T cell features.

At the transcriptional level, *Trem2* knockout increased the expression of the stemness-associated gene Tcf7 and the cytotoxicity-associated gene Gzmb in Cd8^+^ T cells, while reducing the expression of the exhaustion-associated genes Pdcd1 (Pd-1) and Tigit (Fig. 7F). Flow cytometry analysis showed that *Trem2* deletion did not significantly alter the overall proportion of Cd4^+^ T cells but significantly reduced the frequency of Foxp3^+^ Tregs and Pd-1 expression among Cd4^+^ T cells (Fig. 7G to I), while increasing the proportion of naive Cd4^+^ T cells (Sell^+^Cd4^+^; Fig. S10F). In addition, *Trem2^−/−^* tumors showed increased proportions of total Cd8^+^ T cells and naive Cd8^+^ T cells (Sell^+^Cd8^+^ T cells), together with a reduced proportion of Pd-1^+^ exhausted Cd8^+^ T cells (Fig. 7J). Although Gzmb expression showed a higher trend in *Trem2^−/−^* tumors, this difference did not reach statistical significance in the flow cytometry analysis (Fig. 7J).

To determine whether these immune changes were attributable to macrophage-derived Trem2, we performed macrophage-targeted *Trem2* silencing in ICC mice. Consistent with the scRNA-seq findings, *Trem2* silencing significantly reduced the proportion of Foxp3^+^ Tregs and Pd-1^+^ Cd8^+^ T cells, whereas the overall Cd4^+^/Cd8^+^ T cell ratio was not significantly changed (Fig. S10G). Several other T cell subsets did not reach statistical significance, possibly because of variability in the efficiency of AAV8-mediated *Trem2* silencing.

We next characterized 2 distinct Treg subsets in the ICC TME. TNFRSF4^hi^ Tregs (CD4T_Treg_TNFRSF4) showed a stronger association with TREM2^+^ macrophages than TIGIT^hi^ Tregs (CD4T_Treg_TIGIT) (Fig. S12A). Ligand–receptor analysis suggested distinct communication patterns between TREM2^+^ macrophages and TNFRSF4^hi^ Tregs, including CCL3L1–CCR1, CCL8–CCR1/CCR5, PD-L1–PD-1 (CD274–PDCD1), and CD80–CTLA-4 interactions (Fig. S12B). Compared with TIGIT^hi^ Tregs, TNFRSF4^hi^ Tregs expressed higher levels of activation-associated genes, including TNFRSF18, IL2RA, TNFRSF9, and CCR8 (Fig. S12C and D), suggesting a more activated Treg phenotype.

Previous work by Itahashi et al. [56] identified basic leucine zipper ATF-like transcription factor (BATF) as a regulator of Treg activation. By integrating their Treg scRNA-seq dataset (GSE211068; Table S6) with our data, we found that TNFRSF4^hi^ Tregs exhibited higher BATF expression and overlapped with the TME_eTreg compartment, which represents effector Tregs in the TME (Fig. S12E). Pseudotime analysis showed a gradual increase in TNFRSF8, REL, and BATF expression along a transcriptional trajectory from naive CD4^+^ T cells toward activated and suppressive Treg states, including TNFRSF4^hi^ and TIGIT^hi^ Tregs. TCR-signaling, effector, and canonical Treg signatures also increased along this trajectory (Fig. S12F and G). These results support a continuum of transcriptional states associated with Treg activation, although they do not by themselves establish direct conversion of naive CD4^+^ T cells into Tregs.

To further investigate the effects of TREM2^+^ macrophages on CD4^+^ T cells, particularly Tregs, we isolated Trem2^+^ and Trem2^−^ macrophages from ICC tumors of Yap-Akt-induced ICC mice and cocultured them with naive Cd4^+^ T cells isolated from the spleens of healthy mice (Fig. 8A and Fig. S12H). Under TGF-β-supplemented conditions, both Trem2^+^ and Trem2^−^ macrophages promoted the differentiation of naive Cd4^+^ T cells toward a Foxp3^+^ Treg phenotype, whereas Trem2^+^ macrophages exerted a significantly stronger effect (Fig. 8B). In the absence of exogenous TGF-β, only Trem2^+^ macrophages substantially increased the proportion of Foxp3^+^ T cells (Fig. 8C), suggesting that TREM2^+^ macrophages can promote Treg induction through mechanisms that are at least partly independent of exogenous TGF-β.

**Fig. 8.**
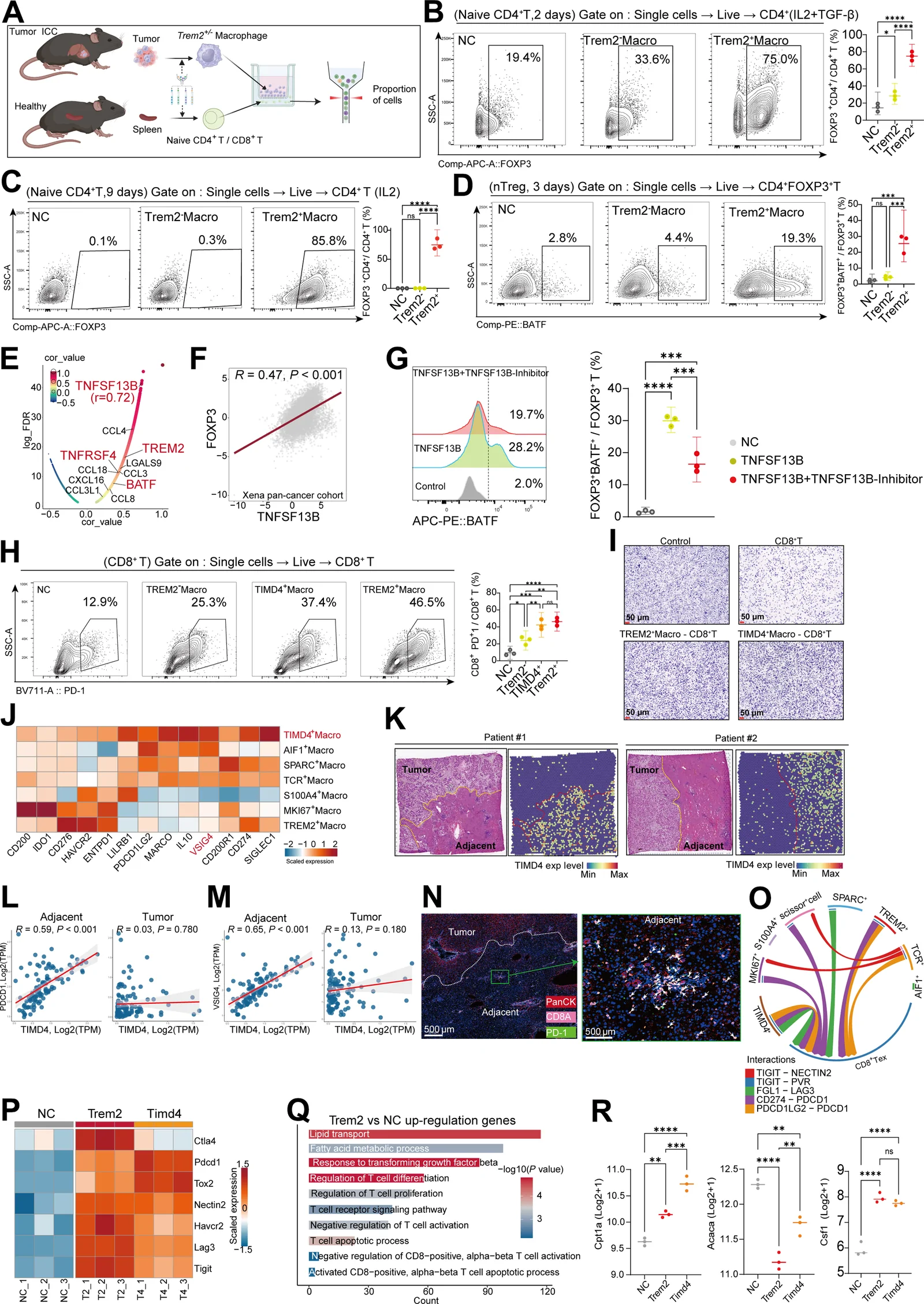
TREM2^+^ macrophages promote immunosuppressive microenvironment in ICC by regulating CD8^+^ T cell exhaustion and Treg development. (A) Schematic diagram of the experimental workflow for macrophage and T cell coculture assays. Macrophage subsets were isolated from ICC tumors, and naive Cd4^+^ T cells or Cd8^+^ T cells were isolated from the spleens of healthy mice for subsequent coculture experiments. (B) Flow cytometry analysis of Foxp3 expression in naive Cd4^+^ T cells cocultured with sorted Trem2^−^ macrophages or Trem2^+^ macrophages for 2 days in the presence of IL-2 and TGF-β. Cells were gated sequentially on single cells, live cells, and Cd4^+^ T cells. Representative flow cytometry plots and quantification of Foxp3^+^ cells among Cd4^+^ T cells are shown (^*^P < 0.05, ^****^P < 0.0001; one-way ANOVA by Dunnett’s test). (C) Flow cytometry analysis of Foxp3 expression in naive Cd4^+^ T cells cocultured with sorted Trem2^−^ macrophages or Trem2^+^ macrophages for 9 days in the presence of IL-2 but without exogenous TGF-β. Cells were gated sequentially on single cells, live cells, and Cd4^+^ T cells. Representative flow cytometry plots and quantification of Foxp3^+^ cells among Cd4^+^ T cells are shown (^*^P < 0.05, ^****^P < 0.0001, ns, not significant; one-way ANOVA by Dunnett’s test). (D) Batf expression in nTreg cells cocultured with macrophages (^***^P < 0.001; ns, not significant; one-way ANOVA by Dunnett’s test). (E) Spearman correlation analysis between Treg signatures and immune-related genes in the FuDan-ICC cohort (Spearman test). (F) Correlation of TNFSF13B with FOXP3 expression in the Xena pan-cancer cohort (Spearman test). (G) Batf expression in nTreg cells in indicated groups. Flow cytometry analysis of Batf expression in Treg cells treated with recombinant TNFSF13B in the presence or absence of a TNFSF13B inhibitor. Representative histogram plots and quantification of Batf^+^ cells among Foxp3^+^ T cells are shown (^***^P < 0.001, ^****^P < 0.0001; one-way ANOVA by Dunnett’s test). (H) Pd-1 expression in Cd8^+^ T cells stimulated by macrophage subtypes (^*^P < 0.05; ^**^P < 0.01; ^***^P < 0.001; ^****^P < 0.0001, ns, not significant; one-way ANOVA by Dunnett’s test). (I) In vitro cytotoxicity assay assessing the killing activity of OT-1 Cd8^+^ T cells against OVA-expressing ICC tumor cells after coculture with macrophage subsets. Tumor cells were visualized by crystal violet staining. Representative images of residual OVA-ICC tumor cells are shown for the control, Cd8^+^ T cell alone, Trem2^+^ macrophage–Cd8^+^ T cell coculture, and Timd4^+^ macrophage–Cd8^+^ T cell coculture groups. (J) Heatmap of immunosuppressive molecule expression in macrophage subcell types (scaled value). (K) Spatial transcriptomics of TIMD4 expression and localization in tumor tissue of 2 ICC patients. (L and M) Spearman correlation of TIMD4 with immunosuppressive markers [PDCD1 (L), VSIG4 (M)] in tumor and adjacent tissues (EHBH-ICC cohort). (N) Multiplex immunofluorescence staining of ICC tissue showed the spatial distribution of PanCK, CD8A, and PD-1 signals in tumor and adjacent tissues (PanCK marks epithelial tumor cells, CD8A marks CD8^+^ T cells, and PD-1 marks PD-1^+^ cells; the white dashed line indicates the tumor-adjacent tissue boundary; Magnified images of the boxed adjacent-tissue region and single-channel images are shown). (O) Ligand–receptor interaction analysis between macrophage subsets and exhausted CD8^+^ T cells in adjacent tissues based on scRNA-seq data. The chord diagram shows predicted immune checkpoint ligand–receptor pairs. (P) Bulk RNA-seq analysis of Cd8^+^ T cells cocultured with sorted Trem2^+^ macrophages or Timd4^+^ macrophages. Heatmap shows scaled mRNA expression levels of selected inhibitory receptor- and exhaustion-related genes compared with control Cd8^+^ T cells. (Q) GO enrichment of up-regulated DEGs in Cd8^+^ T cells cocultured with Trem2^+^ macrophages. (R) Expression levels of fatty acid metabolism-related genes and *Csf1* in Cd8^+^ T cells under coculture with macrophages (the mRNA expression levels were log2-transformed; ^**^P < 0.01, ^***^P < 0.001, ^****^P < 0.0001, ns, not significant; one-way ANOVA by Dunnett’s test). Abbreviations: ICC, intrahepatic cholangiocarcinoma; WT, wild type; TREM2, triggering receptor expressed on myeloid cells 2; TIMD4, T cell immunoglobulin and mucin domain-containing protein 4; TGF-β, transforming growth factor-β; IL-2, interleukin-2; FOXP3, forkhead box P3; BATF, basic leucine zipper ATF-like transcription factor; Treg, regulatory T cell; nTreg, natural regulatory T cell; TNFSF13B, tumor necrosis factor ligand superfamily member 13B; PD-1, programmed cell death protein 1; OVA, ovalbumin; mIF, multiplex immunofluorescence; PanCK, pan-cytokeratin; RNA-seq, RNA sequencing; DEG, differentially expressed gene; GO, Gene Ontology; TPM, transcripts per million; TME, tumor microenvironment.

Because TREM2^+^ macrophages were associated with activated Treg populations in human ICC, we next assessed BATF, a transcriptional regulator linked to Treg activation. Trem2^+^ macrophages markedly increased BATF expression in naive Tregs, whereas Trem2^−^ macrophages had no significant effect (Fig. 8D). These findings indicate that TREM2^+^ macrophages promote the acquisition of a Treg phenotype by naive CD4^+^ T cells and enhance Treg activation-associated features.

To identify potential mediators of Treg activation, we analyzed ligand–receptor interactions between TREM2^+^ macrophages and TNFRSF4^hi^ or TIGIT^hi^ Tregs. The TNFSF13B–TNFRSF13B pair was preferentially predicted in the interaction between Trem2^+^ macrophages and TNFRSF4^hi^ Tregs, with limited interaction strength toward naive CD4^+^ T cells (Fig. S12I). In an independent ICC cohort, TNFSF13B expression was positively correlated with the TNFRSF4^hi^ Treg signature (*r* = 0.72), as well as with TREM2 (*r* = 0.52), TNFRSF4 (*r* = 0.43), and BATF (*r* = 0.39; Fig. 8E). Similar positive associations between TNFSF13B and FOXP3 or CCR8 were observed in the Xena pan-cancer cohort (Fig. 8F and Fig. S12J). Functionally, recombinant TNFSF13B increased BATF expression in naive Tregs, whereas this effect was attenuated by a TNFSF13B-targeting inhibitor (Fig. 8G). These results support TNFSF13B as a candidate macrophage-derived mediator that contributes to Treg activation downstream of TREM2^+^ macrophages.

Given the central role of cytotoxic CD8^+^ T cells in antitumor immunity, we next investigated the interactions between macrophage subsets and exhausted CD8^+^ T cells in ICC tumors. TREM2^+^ macrophages showed strong predicted interactions with CD8^+^ Tex cells in tumor tissues (Fig. S13A and B). Ligand–receptor analysis identified several potential interaction axes, including immune checkpoint pathways such as PD-L1–PD-1 (CD274–PDCD1), PD-L2–PD-1 (PDCD1LG2–PDCD1), NECTIN2–TIGIT, and FGL1–LAG3, as well as chemokine pathways including CXCL9–CXCR3, CXCL10–CXCR3, and CXCL16–CXCR6 (Fig. S13C and D). These predicted interactions suggest that TREM2^+^ macrophages may influence both the recruitment and the exhaustion-associated state of CD8^+^ T cells.

To experimentally assess this possibility, we isolated naive Cd8^+^ T cells from the spleens of healthy mice and cocultured them with Trem2^+^ or Trem2^−^ macrophages isolated from ICC tumors. Both macrophage populations increased Pd-1 expression in Cd8^+^ T cells, but the effect was stronger in the presence of Trem2^+^ macrophages (Fig. 8H and I). We also examined TIMD4^+^ macrophages, a Trem2^−^ macrophage subset that expressed several immunosuppressive molecules, including VSIG4 and SIGLEC1 and CD274 (PD-L1 [57,58]; Fig. 8J). TIMD4^+^ macrophages were enriched in peritumoral and adjacent regions rather than being restricted to the tumor core (Figs. 1L and 8K). Consistent with their immunosuppressive profile, Timd4^+^ macrophages also induced Pd-1 expression in Cd8^+^ T cells, although less strongly than TREM2^+^ macrophages (Fig. 8H). This observation is consistent with previous findings reported by Chow et al. [31]. In addition, coculture of OT-1 Cd8^+^ T cells with Timd4^+^ or Trem2^+^ macrophages markedly reduced their cytotoxicity against OVA-expressing ICC cells compared with the control condition (Fig. 8I). These findings indicate that both macrophage subsets can suppress CD8^+^ T cell effector function, with TREM2^+^ macrophages exerting a stronger effect in the in vitro assay.

Intriguingly, TIMD4^+^ macrophages showed no correlation with CD8^+^ Tex in tumor cores, but strongly associated with PD-1^+^ CD8^+^ T cells in peritumor regions (Fig. 8L and M and Fig. S13E). In addition, the peritumoral zones contained abundant PD-1^+^ CD8^+^ T cells (Fig. 8N). CellphoneDB analysis in both regions revealed the prominent immunosuppressive features of ligand–receptor pairs between TIMD4^+^ macrophages and CD8^+^ Tex (Fig. 8O), with peritumor CD8^+^ Tex-associated genes (VSIG4 [58], PSAP [59], as previous studies) predominantly expressed by TIMD4^+^ macrophages (Fig. S13F). These findings suggested that TIMD4^+^ macrophages primarily drove the exhaustion of CD8^+^ T cells at the peritumor and adjacent regions.

We next cocultured Cd8^+^ T cells with Trem2^+^ or Timd4^+^ macrophages and performed bulk RNA sequencing (RNA-seq). Principal component analysis and transcriptomic profiling revealed distinct expression patterns among the control, Trem2^+^, and Timd4^+^ macrophage coculture groups (Fig. 8P and Fig. S13G; DEGs are listed in Tables S8 and S9). Compared with control Cd8^+^ T cells, macrophage-exposed Cd8^+^ T cells showed increased expression of exhaustion-associated genes and enrichment of pathways related to lipid transport, fatty-acid metabolism, TGF-β responses, negative regulation of T cell activation and proliferation, and T cell apoptotic processes (Fig. 8Q and Fig. S13H). Consistent with the enrichment analysis, Cpt1a expression was increased whereas Acaca expression was reduced after macrophage coculture, suggesting enhanced fatty-acid utilization (Fig. 8R). Csf1 expression was also increased in macrophage-exposed Cd8^+^ T cells, raising the possibility of a reciprocal feedback loop between exhausted Cd8^+^ T cells and Trem2^+^/Timd4^+^ macrophages. This potential interaction requires further experimental validation.

Collectively, these findings support spatially compartmentalized immunoregulatory functions of macrophage subsets in ICC. TREM2^+^ macrophages promote Treg induction and activation and are strongly associated with the exhaustion-associated state and impaired cytotoxicity of CD8^+^ T cells, particularly within tumor tissues. TIMD4^+^ macrophages also suppress CD8^+^ T cell function but are preferentially associated with PD-1^+^CD8^+^ T cells in peritumoral and adjacent regions. Notably, these peritumoral and adjacent regions contain few or no tumor cells but harbor abundant PD-1^+^CD8^+^ T cells, suggesting that TIMD4^+^ macrophages may contribute to CD8^+^ T cell dysfunction through local or paracrine immunosuppressive signals. Direct evidence for long-range secretory mechanisms, however, remains to be established.

### Therapeutic potential of TREM2 and TIMD4 blockade for enhancing anti-PD-1 efficacy against ICC

Given the immunosuppressive functions associated with TREM2^+^ and TIMD4^+^ macrophages, we initially hypothesized that CSF1R blockade might deplete these macrophage populations and improve the response to immunotherapy. To test this hypothesis, Kras-P19-induced ICC mice were treated with an anti-Csf1r antibody every 3 days for 6 cycles, beginning 3 weeks after ICC induction (Fig. 9A). Unexpectedly, anti-Csf1r antibody treatment increased liver tumor burden and liver weight compared with the isotype control (Fig. 9B and C). To determine whether this effect was specific to the Kras-P19 model, survival was also assessed in Yap-Akt-induced ICC mice. Anti-Csf1r antibody treatment did not improve survival in Yap-Akt-induced ICC mice and significantly shortened survival in Kras-P19-induced ICC mice (Fig. 9D and E).

**Fig. 9.**
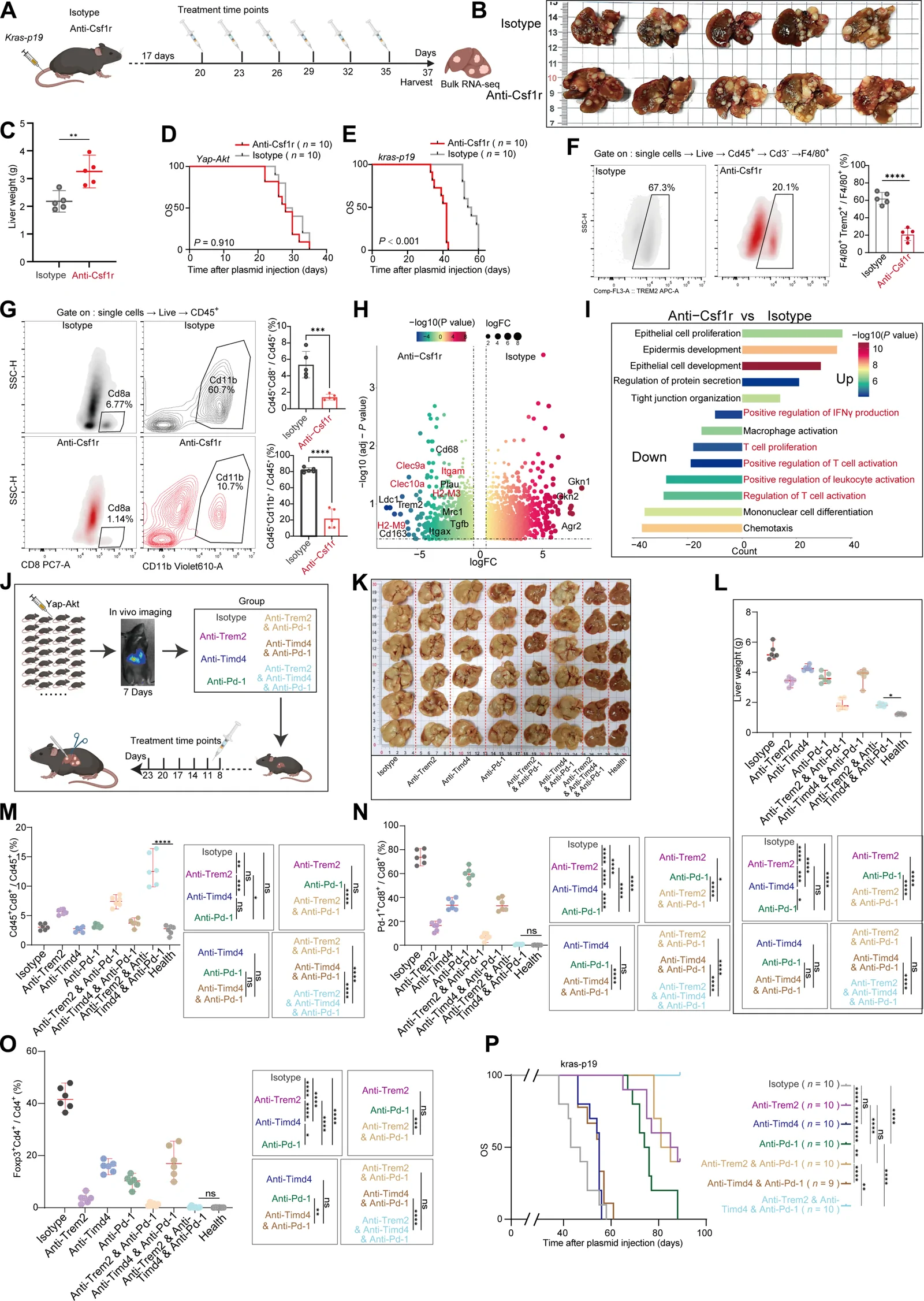
Targeting Trem2 and Timd4 to enhance anti-Pd-1 efficacy in ICC. (A) Workflow diagram of the anti-Csf1r antibody treatment in Kras-P19-induced ICC mouse models. After 6 doses of treatment, tumor tissues were collected for bulk RNA-seq. (B and C) Morphology of tumors in anti-Csf1r antibody treated versus control groups (^**^P < 0.01; Mann–Whitney *U* test). (D and E) Survival curves (Kaplan–Meier) of Yap-Akt-induced and Kras-P19-induced ICC mice treated with anti-Csf1r antibody versus controls. (F) Proportions of Trem2^+^ macrophages among total macrophages in tumor tissues from anti-Csf1r antibody-treated and control mice following 6 anti-Csf1r antibody treatments, as determined by flow cytometry (^****^P < 0.0001; Mann–Whitney *U* test). (G) Cd8^+^ and Cd11b^+^ cell ratios in anti-Csf1r antibody treated versus control mice (the proportion of target cells among total leukocytes; ^***^P < 0.001; ^****^P < 0.0001; Mann–Whitney *U* test). (H and I) Volcano plot (H) and GO enrichment (I) of DEGs in anti-Csf1r antibody treated tumors. (J) Workflow of therapeutic in Yap-Akt-induced ICC mice. One hundred C57BL/6 mice were used to establish ICC model via hydrodynamic tail vein injection of the Yap-Akt-SB100 plasmid. On day 7, tumor burden was assessed using IVIS optical imaging. Forty-eight mice with comparable total fluorescence values were selected and evenly randomized into 8 groups, ensuring no statistically significant difference in initial tumor burden among groups. The mice were then treated every 3 days for a total of 6 times. Two days after the final treatment, the mice were fasted for 6 h before tumor tissues were harvested for flow cytometric analysis. (K and L) Tumor morphology and the liver weight across treatment groups. (M to O) Flow cytometric analysis of tumor-infiltrating immune-cell populations in mice treated with isotype control, anti-Trem2, anti-Timd4, anti-Pd-1, anti-Trem2 plus anti-Pd-1, anti-Timd4 plus anti-Pd-1, anti-Trem2 plus anti-Timd4, or anti-Trem2 plus anti-Timd4 and anti-Pd-1 antibodies, as indicated in (J). (M) Proportion of Cd45^+^Cd8^+^ T cells among total Cd45^+^ cells. (N) Proportion of Pd-1^+^ cells among Cd8^+^ T cells. (O) Proportion of Foxp3^+^ cells among Cd4^+^ T cells. Healthy mice were included as a reference group (^*^P < 0.05; ^**^P < 0.01; ^***^P < 0.001; ^****^P < 0.0001; ns, not significant; one-way ANOVA followed by Tukey’s multiple-comparisons test). (P) Survival curves (Kaplan–Meier) of Kras-p19-induced ICC mice under therapeutic interventions. Abbreviations: ICC, intrahepatic cholangiocarcinoma; GO, Gene Ontology; DEG, differentially expressed gene; SB100, Sleeping beauty transposon 100.

Bulk RNA-seq and flow cytometry analyses provided potential explanations for this unexpected response. Principal component analysis showed clear separation between anti-Csf1r antibody-treated and isotype-treated tumors, with relatively limited within-group variation (Fig. S14A). Anti-Csf1r antibody treatment reduced the abundance of macrophage and monocyte populations, including cells expressing Trem2, Itgam, Mrc1, and Cd163 (Fig. 9F to H). However, it also reduced the expression of cDC1-associated genes, including Clec9a, H2-M3, and H2-M9 (additional DEGs are provided in the Table S10; Fig. 9H). Consistent with these changes, gene-set enrichment analysis showed reduced programs related to T cell activation, proliferation, and IFN-γ production (Fig. 9I), and flow cytometry demonstrated a decrease in intratumoral CD8^+^ T cell abundance after anti-Csf1r antibody treatment (Fig. 9G). These findings suggest that the anti-Csf1r antibody-induced loss of macrophages may be accompanied by impaired cDC1-associated and CD8^+^ T cell responses, potentially offsetting or reversing the expected therapeutic benefit.

In agreement with this interpretation, CSF1R expression was associated with favorable prognosis in patients with ICC (Fig. S14B). Previous studies have also implicated CSF1R signaling in dendritic-cell development and shown that CSF1R inhibition can reduce dendritic-cell populations in the blood and tumor-draining lymph nodes [60]. In addition, CSF1R deficiency may indirectly impair dendritic-cell regeneration by disrupting the homeostasis of tissue-resident macrophages [61]. These observations indicate that broad CSF1R blockade may not be an optimal strategy for macrophage targeting in ICC. We therefore evaluated direct blockade of TREM2 and TIMD4, alone or in combination with immune-checkpoint inhibition.

To minimize differences in baseline tumor burden, 100 ICC-bearing mice were ranked according to their baseline bioluminescence signals. The 42 mice with intermediate fluorescence values were selected and randomly assigned to 7 treatment groups, together with a nontumor-bearing control group. Treatments were administered every 3 days for 3 weeks, for a total of 6 treatment cycles, after which liver tumor burden and immune-cell composition were assessed by liver-weight measurement, bioluminescence imaging, and flow cytometry (Fig. 9J and Fig. S14C).

Among the single-agent treatments, anti-Trem2 and anti-Pd-1 antibodies produced the greatest reductions in tumor burden and showed comparable efficacy relative to the isotype control. Anti-Timd4 antibody monotherapy also reduced tumor burden, although its effect was less pronounced (Fig. 9K and L). Combining anti-Trem2 antibody with anti-Pd-1 further reduced tumor burden compared with either monotherapy. In contrast, the anti-Timd4 plus anti-Pd-1 antibodies combination did not produce a significant additive effect compared with the corresponding monotherapies. The triple combination of anti-Trem2, anti-Timd4, and anti-Pd-1 antibodies did not further reduce tumor burden compared with the anti-Trem2 and anti-Pd-1 antibodies combination (Fig. 9K and L). Thus, under these experimental conditions, the main contribution to tumor control appeared to come from Trem2 blockade and anti-Pd-1 treatment, whereas the additional benefit of Timd4 blockade was limited.

Flow cytometry analysis showed that anti-Trem2 antibody monotherapy significantly increased the proportion of intratumoral CD8^+^ T cells, whereas anti-Timd4 antibody monotherapy did not produce a comparable increase (Fig. 9M and Fig. S14D). The anti-Trem2 plus anti-Pd-1 antibodies combination further increased CD8^+^ T cell infiltration compared with anti-Pd-1 antibody monotherapy, although this increase was not significantly greater than that observed with anti-Trem2 antibody monotherapy. The triple combination yielded the highest Cd8^+^ T cell proportion among the treatment groups (Fig. 9M). Anti-Trem2 antibody markedly reduced the proportion of Pd-1^+^ Cd8^+^ T cells, whereas anti-Timd4 antibody also reduced this exhaustion-associated population without significantly increasing total Cd8^+^ T cell infiltration (Fig. 9M and N and Fig. S14D and E). The anti-Timd4 plus anti-Pd-1 antibodies combination did not further reduce the proportion of Pd-1^+^ Cd8^+^ T cells compared with anti-Timd4 antibody monotherapy. The triple combination produced the strongest reduction in Pd-1^+^ Cd8^+^ T cells (Fig. 9N). This result may reflect the combined effects of macrophage targeting and tumor control; however, the mechanism underlying the apparent superiority of the triple combination requires further investigation.

Because TREM2^+^ macrophages promoted Treg induction in vitro, we also examined Treg abundance in treated tumors. Tregs accounted for approximately 40% of CD4^+^ T cells in the tumors of control mice and were significantly reduced by anti-Trem2 antibody monotherapy (Fig. 9O and Fig. S14F). Anti-Timd4 and anti-Pd-1 antibodies monotherapies also reduced Treg abundance, indicating that the regulation of Tregs was not specific to Trem2 blockade under these conditions.

Anti-Trem2 antibody and anti-Pd-1 antibody monotherapies significantly reduced the proportion of Ly6C^+^ monocytes, but their combination did not produce an additional reduction (Fig. S14G and H). The reduced monocyte abundance may reflect impaired monocyte recruitment or altered monocyte survival after TREM2 blockade. Anti-Pd-1-mediated changes in CD8^+^ T cell activity may also indirectly affect monocyte recruitment. However, the precise mechanisms responsible for these changes remain to be determined.

We next evaluated treatment-associated survival in Kras-P19- and Yap-Akt-induced ICC models using the same treatment schedules. In Kras-P19-induced ICC mice, the triple combination produced the greatest survival benefit, followed by the anti-Trem2 plus anti-Pd-1 antibodies combination and anti-Trem2 antibody monotherapy; both anti-Trem2-containing regimens outperformed anti-Pd-1 monotherapy (Fig. 9P). Anti-Timd4 antibody monotherapy and the anti-Timd4 plus anti-Pd-1 antibodies combination showed limited overall survival benefits, although transient responses were observed during the early to intermediate stages of tumor development. These findings suggest that the therapeutic effect of TIMD4 blockade may depend on tumor model and disease stage, rather than being uniformly beneficial.

In Yap-Akt-induced ICC mice, the triple combination again produced the longest survival, followed by the anti-Trem2 plus anti-Pd-1 antibodies combination (Fig. S14I). In contrast to the Kras-P19 model, anti-Timd4 antibody showed a more evident benefit when combined with anti-Pd-1 antibody, although anti-Timd4 antibody monotherapy remained the least effective treatment. Together, these results indicate that TIMD4 blockade may have context-dependent activity, whereas TREM2 blockade shows a more consistent therapeutic effect across the 2 ICC models.

We also assessed the in vivo safety of the therapeutic antibodies. Healthy mice were treated with the indicated agents and compared with saline-treated controls. Safety was evaluated by complete blood counts, liver and renal function tests (Fig. S15A), and hematoxylin and eosin staining of major organs (Fig. S15B). Anti-Fap antibody treatment was associated with a mild reduction in platelet counts, whereas anti-Timd4 antibody treatment affected red blood cell and platelet counts. Anti-Trem2 antibody treatment did not significantly alter these parameters (Fig. S15A). No obvious histopathological abnormalities were observed in the liver, lung, spleen, heart, or kidney across the treatment groups (Fig. S15B). These results suggest that the tested treatments had no major histological toxicity under the conditions examined, although the hematological changes associated with anti-Fap antibody and anti-Timd4 antibody warrant further evaluation.

Collectively, these findings support the therapeutic activity of Trem2 blockade and its ability to enhance anti-Pd-1-mediated tumor control in ICC. Although Timd4 blockade reduced Treg abundance and the proportion of Pd-1^+^ Cd8^+^ T cells, it did not consistently improve the efficacy of anti-Pd-1 antibody, particularly in the Kras-P19-induced ICC model. The therapeutic effects of Timd4 blockade were dependent on the ICC model and treatment context. These results suggest that TREM2 may represent a more broadly applicable macrophage target than TIMD4 for combination treatment with PD-1 blockade, whereas the clinical utility of TIMD4 targeting requires further investigation.

## Discussion

Because of its insidious onset, atypical clinical manifestations, and particularly immunosuppressive microenvironment and aggressive tumor biology, ICC always inflicts patients with a dismal prognosis, despite emerging immunotherapies and targeted agents. Here, we identified TREM2^+^ macrophages as a pivotal regulator of ICC progression, and their infiltration was correlated with the advanced stage, metastasis, and CD8^+^ T cell exhaustion. These findings provide new insights into potential therapeutic strategies for ICC.

The monocyte–macrophage cells, critical for hepatic homeostasis (e.g., pathogen clearance and immune tolerance [8]), undergo profound numerical and functional remodeling in ICC. Tumor-educated monocyte-derived macrophages can develop into TAM phenotypes [9], thus driving angiogenesis, cancer stemness, T cell exhaustion, and immunosuppressive crosstalk with stromal cells [5]. Here, we clarified the heterogeneity, spatial distribution, and functional specialization of TAM subsets in ICC and adjacent tissues via scRNA-seq and validated these features through in vitro/vivo experiments (Fig. 10).

**Fig. 10.**
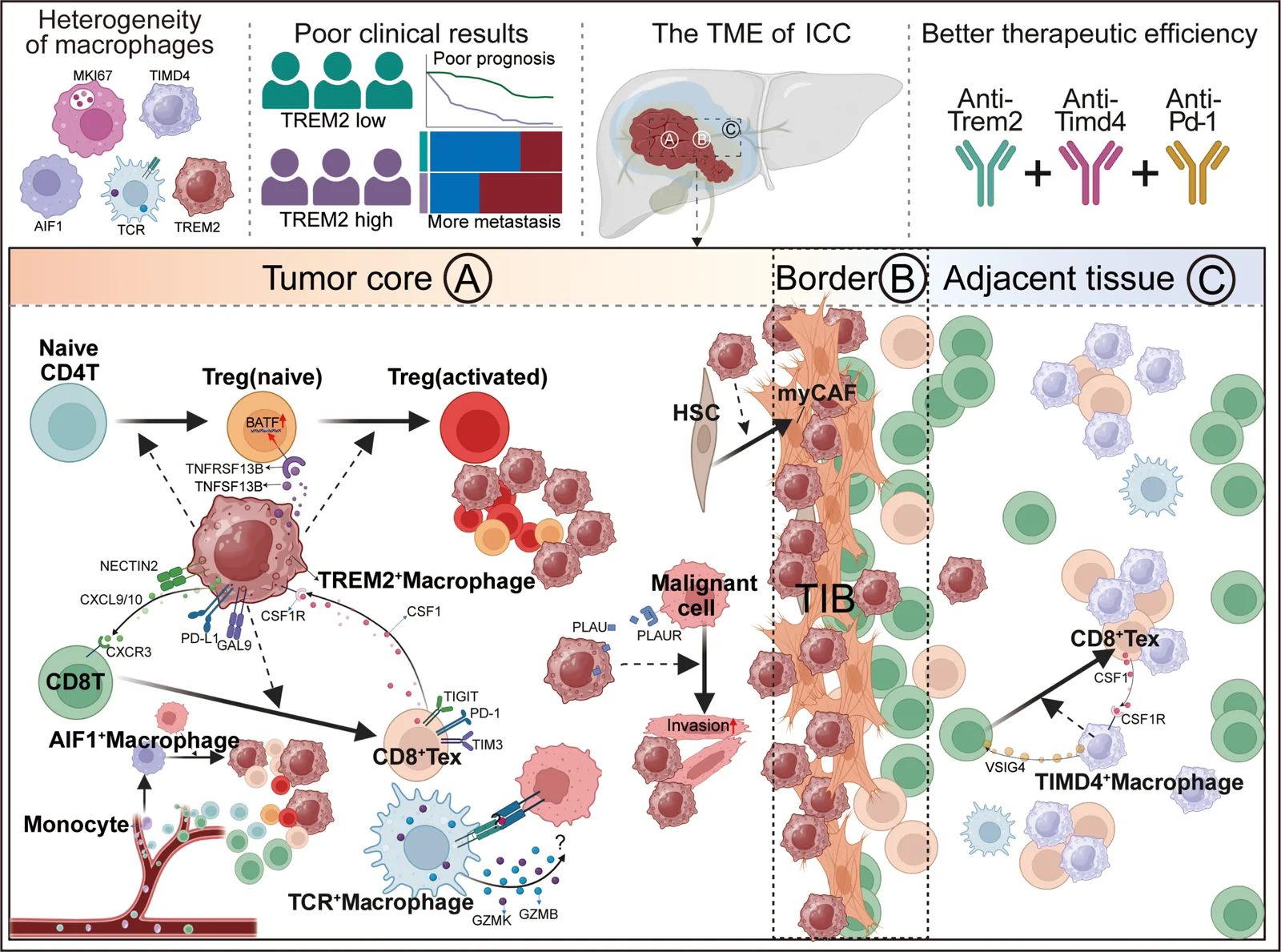
Spatial organization and immunosuppressive roles of macrophage subtypes in the ICC tumor microenvironment. This study identifies 5 stable macrophage subcell types in ICC, among which TREM2^+^ macrophages are considerably associated with poor ICC patient prognosis. Mechanistically, in the ICC TME, TREM2^+^ macrophages likely derive from blood monocytes and differentiate through an AIF1^+^ macrophage intermediate. On one hand, TREM2^+^ macrophages promote the differentiation of naive CD4^+^ T cells into Tregs via TNFSF13B and further activate naive Tregs; on the other hand, they drive CD8^+^ T cell exhaustion. Exhausted CD8^+^ T cells in turn support TREM2^+^ macrophage survival through CSF1. Interestingly, TCR^+^ macrophages within tumors may directly kill tumor cells via molecules such as GZMB. Additionally, TREM2^+^ macrophages at the tumor boundary facilitate the transition of HSC into myCAFs, thereby forming an immune barrier that impedes intratumoral infiltration of CD8^+^ T cells. Furthermore, TREM2^+^ macrophages promote tumor invasion and metastasis through the PLAU–PLAUR axis. Surprisingly, TIMD4^+^ macrophages in peritumoral normal liver tissue also contribute to CD8^+^ T cell exhaustion. Finally, combining anti-Trem2, anti-Timd4, and anti-Pd-1 therapies revealed that anti-Trem2 alone exerts a monotherapeutic effect and synergistically enhances the efficacy of anti-Pd-1 treatment. Abbreviations: ICC, intrahepatic cholangiocarcinoma; TME, tumor microenvironment; HSC, hepatic stellate cells; myCAF, myofibroblastic cancer-associated fibroblasts.

scRNA-seq analysis revealed that TREM2^+^ macrophages were enriched in tumor cores and characterized by tumor-promoting properties. Although TIMD4^+^ macrophages shared Kupffer cell-like features, they exhibited distinct transcriptional and functional profiles in peritumoral regions, a pattern consistently observed across different ICC mouse models. We propose a differentiation trajectory of TIMD4^+^ macrophages: circulating monocytes differentiate into AIF1^+^ macrophages, which grow into Kupffer-like cells or monocyte-derived macrophages under homeostasis. TREM2-driven reprogramming takes place during tumorigenesis across all subsets, marked by up-regulation of TAM-associated genes. Although lineage-tracing evidence remains pending, functional assays confirmed that TREM2^+^ macrophages can promote tumor invasion, tissue remodeling, and immunosuppression.

Genetic ablation (*Trem2^−/−^*) or macrophage-specific silencing (AAV8-*F4/80^Cre^*-sh-*Trem2*) prolonged the survival and reduced the tumor burden in ICC mice, corroborated by the prognostic significance of TREM2 in clinical cohorts. Single-cell comparisons between Trem2 and WT tumors revealed, and flow cytometry validated the diminishment of fibroblasts, Tregs, and exhausted CD8^+^ T cells post-*Trem2* deletion. Despite lacking *F4/80^Cre^-Trem2^f/f^* comparisons, concordant findings across mouse models supported our robust conclusions.

Fibrotic stroma, a key immunotherapy barrier in ICC [62], was reinforced as TREM2^+^ macrophages facilitated the transition of fibroblasts into POSTN^+^ CAFs. The spatial analysis identified that peritumor TREM2^+^ macrophages and POSTN^+^ CAFs can form physical barriers to keep away CD8^+^ T cell infiltration. Concurrently, TREM2^+^ macrophages triggered Treg induction/activation (via TNFSF13B) and collaborated with TIMD4^+^ macrophages to exhaust CD8^+^ T cells, sustaining immunosuppression. In addition to promoting tumor progression by modulating the immune microenvironment, TREM2^+^ macrophages also directly enhance tumor cell invasion and migration through the PLAU–PLAUR axis.

The combination of anti-Trem2 and Timd4 with anti-Pd-1 demonstrated an enhanced efficacy, with anti-Trem2 synergizing strongly with anti-Pd-1 to reduce CD8^+^ T cell exhaustion and Treg proportions. The efficacy of anti-Timd4 varied with molecular subtype, highlighting the context-dependent roles of Timd4.

The limitations of this study include incomplete lineage-tracing validation and lack of large-scale clinical validation. Furthermore, this study did not delve deeper into the mechanisms by which PLAUR promotes tumor progression and TNFSF13B facilitates the activation of Treg cells. Nevertheless, these findings offer transformative insights for ICC treatment and microenvironment-targeted strategies across malignancies. Finally, anti-Trem2 or anti-Pd-1 coadministration might be adopted as the optimal strategy for ICC driven by Kras mutation. However, its clinical translation will surely take a long road due to the heterogeneous molecular mutations in patients.

## Conclusions

This study provides an atlas of heterogeneous macrophages in the tumor, peritumor, and adjacent regions of ICC. TREM2^+^ and TIMD4^+^ macrophages are key contributors to the immunosuppressive microenvironment in ICC. Anti-Trem2 can boost the effectiveness of anti-Pd-1 therapy.

## Ethical Approval

This study was approved by The Third Affiliated Hospital of Naval Medical University Institutional Ethics Committee (permit number: EHBHKY2020-K-022). All ICC patient samples involved in this study were obtained only after the patients had carefully read the relevant information and provided written informed consent. All animal experiments were reviewed and approved by the Ethics Committee and Animal Welfare Committee of Nanjing Drum Tower Hospital, affiliated with the Medical School of Nanjing University (permit number: 2022AE01013). All animal-related procedures were performed in accordance with the institutional guidelines for animal welfare, and every effort was made to minimize the number of animals used. All procedures involving animals were preapproved and aligned with the institutional guidelines for animal welfare, and the experiments were carefully designed to reduce the number of animals utilized.

## Data Availability

All data generated in this study, excluding those obtained from public databases, are available upon reasonable request to the corresponding authors. Open-access datasets are provided in Table S6.
