# Supplementary material for: Targeting TREM2+ and TIMD4+ Macrophages Reprograms the Tumor Microenvironment and Enhances PD-1 Blockade Efficacy in Intrahepatic Cholangiocarcinoma
Source: Cancer Commun (Lond). 2026 Oct 9;46:0056. doi: 10.34133/cancomm.0056 (PMC13650241; doi:10.34133/cancomm.0056)
Supplement: Supplementary file 1 [file cancomm.0056.f1.zip › 25-00088-Supplementary Materials-final.docx]

**Supplementary Materials**

**Targeting TREM2⁺ and TIMD4⁺ macrophages reprograms the tumor microenvironment and enhances PD-1 blockade efficacy in intrahepatic cholangiocarcinoma**

Yao Li^1,†^, Hongwen Liu^2,†^, Lei Xu^2,†^, Lili Huang^3,†^, Hui Xue^1,†^, Tao Lu^4^, Yuxuan Lin^1^, Liangrui Chen^1^, Jianwei Liu^1^, Shilei Bai^1^, Yong Xia^4^, Lei Wang^2,*^, Hui Dong^4,*^, Feng Shen^3,*^, Kui Wang^1,*^

^1^Department of Hepatic Surgery II, Eastern Hepatobiliary Surgery Hospital, Naval Medical University, Shanghai, P. R. China.

^2^Department of Gastroenterology, Nanjing Drum Tower Hospital, The Affiliated Hospital of Nanjing University Medical School, Nanjing, Jiangsu, P. R. China.

^3^Department of Hepatic Surgery IV, Eastern Hepatobiliary Surgery Hospital, Naval Medical University, Shanghai, P. R. China.

^4^Department of Pathology, Eastern Hepatobiliary Surgery Hospital, Naval Medical University, Shanghai, P. R. China.

^†^Yao Li, Hongwen Liu, Lei Xu, Lili Huang, and Hui Xue contributed equally to this work.

^*^Corresponding author:

Kui Wang; [wangkuiykl@163.com](mailto:wangkuiykl@163.com).

Feng Shen; [shenfengehbh@sina.com](mailto:shenfengehbh@sina.com).

Hui Dong; [huidongwh@126.com](mailto:huidongwh@126.com).

Lei Wang; [leiwang9631@nju.edu.cn](mailto:leiwang9631@nju.edu.cn).

**Supplementary Tables**

**Supplementary Table S1. Clinicopathological characteristics of patients with ICC in this study.**

[Excel]

**Supplementary Table S2. Antibodies used in this study.**

[Excel]

**Supplementary Table S3. Gene signatures used in this study.**

[Excel]

**Supplementary Table S4. Co-expression correlation analysis of TREM2 and POSTN in ICC samples via multiplex immunofluorescence.**

[Excel]

**Supplementary Table S5. Sequences for shRNAs mentioned in this study.**

[Excel]

**Supplementary Table S6. Datasets used in this study.**

[Excel]

**Supplementary Table S7. The top 20 marker genes for macrophage subpopulations.**

[Excel]

**Supplementary Table S8. Differentially expressed genes up-regulated in CD8^+^ T cells following co-culture with Trem2^+^ macrophages.**

[Excel]

**Supplementary Table S9. Differentially expressed genes up-regulated in CD8^+^ T cells following co-culture with Timd4^+^ macrophages.**

[Excel]

**Supplementary Table S10. Anti-Csf1r vs control differentially expressed genes with bulk RNA-seq.**

[Excel]

**Supplementary Figure**

**Supplementary Figure S1. Macrophage abundance is associated with clinical outcome and reveals distinct macrophage subsets and spatial distributions in ICC.**

(A) DEGs analysis between long-term and short-term survivors in the FuDan-ICC-cohort was performed using the limma algorithm, and the identified genes were displayed in a volcano plot. Red dots indicate upregulated genes, blue dots indicate downregulated genes, and gray dots indicate genes without significant changes.
(B) Dot plot showed the hazard ratios of the top 10,000 highly variable genes in the FuDan-ICC-cohort, calculated by univariate Cox proportional hazards regression analysis. Representative myeloid, fibroblast and malignant cell-related genes are labeled.

(C-D) After GO enrichment analysis of the differentially expressed genes upregulated in patients with short survival (Fudan-ICC-cohort), bar plots were used to display epithelial tumor-related terms (C) and myeloid cell-related terms (D). “Count” represents the number of genes involved in the GO enrichment analysis.

(E) UMAP plots of all cells and myeloid cells in scRNA-seq data.
(F) Feature plots showed the expression of representative myeloid lineage markers in the scRNA-seq dataset, including FCN1 for monocytes, FCGR3B for neutrophils, and CLEC9A and CD1C for dendritic cells.
(G) Kaplan-Meier survival analysis of myeloid markers in the FuDan-ICC-cohort.
(H-K) Dot plots (H-I) and GO enrichment (J-K) of genes correlated with exhausted CD8^+^ T cell signatures in FuDan-ICC-cohort (H, J) and EHBH-ICC-cohort (I, K; Spearman test).
(L) Feature plots showed the expression of genes positively correlated with exhausted CD8^+^ T cell signatures in the scRNA-seq dataset, including SAMHD1, MS4A6A, C1QB, C1QC, TNFSF13B, CSF1R, and CD68.
(M) Box plots showed CD68 expression levels in ICC patients with different genetic mutation statuses, including *KRAS, IDH1, TP53, ARID1A,* and *MUC16* mutations, based on bulk RNA-sequencing data. CD68 expression levels are shown as log2 (TPM + 1; ^*^*P* < 0.05, Mann-Whitney U-test).

Abbreviations: ICC, intrahepatic cholangiocarcinoma; DEG, differentially expressed gene; HR, hazard ratio; UMAP, Uniform Manifold Approximation and Projection; GO, Gene Ontology; scRNA-seq, single-cell RNA sequencing; TPM, transcripts per million.

**Supplementary Figure S2. Macrophage abundance is associated with clinical outcome and reveals distinct macrophage subsets and spatial distributions in ICC.**

(A) Followed scRNA-seq analysis, violin plots were used to show the expression levels of CD68, LCK, LAT, and FYN across different macrophage cell subtypes (normalized expression data).
(B) Followed scRNA-seq analysis, umap plots were used to show the expression levels of CD3E, IFNG, GZMA, and GZMB across different macrophage cell subtypes (normalized expression data).
(C) GZMA Secretion levels in TCR^+^ macrophages (CD3^+^CD68^+^), TCR^-^ macrophages (CD3^-^CD68^+^), and effector CD8^+^ T cells (CD44^+^CD8^+^ T cells) were measured by ELISA (one-way ANOVA by Dunnett’s test).
(D) Gating strategy for leukocyte (CD45^+^ cells) flow cytometry in ICC mice.
(E) Cite-seq data showed surface protein expression of CD3E and CD68, with the red circle indicating the co-expression region of both markers. Anti-human CD3E, barcode-ACTTATCTCTGTGTA; anti-human CD68, barcode-TGGACATTCATGATA.
(F) Spatial transcriptomics of TREM2^+^ macrophage distribution in peritumor region.

Abbreviations: scRNA-seq, single-cell RNA sequencing; ICC, intrahepatic cholangiocarcinoma; ELISA, enzyme-linked immunosorbent assay.

**Supplementary Figure S3. Conservation of macrophage cell subtypes across species in the ICC microenvironment.**

(A) Clustering of macrophages based on activity scores of 220 transcription factors (TFs) showed by UMAP plot. TFs activity was calculated using the pySCENIC software, as described in the methods section.

(B-C) Heatmaps showed TFs activity levels across cell subtypes (B, clusters by highly variable genes; C, clusters directly based on TFs activity).

(D) UMAP plot showed Trem2 expression in macrophages from Kras-P19-induced ICC tumors.

(E) Flow cytometry analysis of Trem2^+^ macrophage proportions in Yap-Akt-induced ICC tumor tissue versus normal liver tissue (^**^*P* < 0.01, Mann-Whitney U-test).

(F) Flow cytometry analysis of Trem2^+^ macrophage in Kras-P19-induced ICC tumor tissue versus normal liver tissue (^**^*P* < 0.01, Mann-Whitney U-test).

(G) mIF staining showed Trem2^+^ macrophages in Kras-P19-induced ICC mice versus normal liver.

(H) Heatmap of macrophage cell subtypes similarity between Yap-Akt and Kras-P19-induced ICC mice.

Abbreviations: ICC, intrahepatic cholangiocarcinoma; TF, transcription factor; UMAP, Uniform Manifold Approximation and Projection; mIF, multiplex immunofluorescence.

**Supplementary Figure S4. Transcriptional states and inferred developmental trajectories of macrophages in ICC.**

(A) M1/M2 polarization status of macrophage subtypes (the M1/M2 score was calculated using AUCell. The pentagram symbol represents the geometric center of the M1/M2 scores for this cell subpopulation; signature genes can be found in Supplementary Table S3).
(B) Hierarchical clustering of macrophage subtypes (the top 2,000 variable genes were selected, and the average expression level for each cell type was calculated, followed by hierarchical clustering based on expression patterns).
(C) Volcano plot of DEGs in AIF1^+^ macrophages from tumor versus adjacent tissues.
(D) Volcano plot of DEGs between Timd4^+^ macrophages in Kras-P19-induced ICC (at 8 weeks) and normal liver macrophages

(E) Expression of TIMD4/FCN1 in TIMD4^+^ macrophages from adjacent tissues, tumors, and normal liver by scRNA-seq analysis.

(F) scRNA-seq analysis of Trem2 and Fcn1 expression in Timd4⁺ macrophages from normal and tumors liver tissues at 3, 6, and 8 weeks.

(G) Frequency of Trem2^+^ cell among total cells in peripheral blood monocytes (Blood-mono) and healthy liver-resident Timd4^+^ macrophages before (pre) and after (after) 48 hours of co-culture with ICC tumor cells. Each pair of connected points represents an independent biological replicate (*n* = 3). Data are shown as individual values, and lines connect paired samples from the same experimental batch. Statistical significance between the “after” groups (Blood-mono vs. Healthy-liver) was determined by an unpaired two-tailed Student’s t-test.

(H) The activity of each transcription factor in different macrophage subtypes was inferred using pySCENIC, and the activity levels of these transcription factors in AIF1^+^, TIMD4^+^, TREM2^+^, and healthy macrophages were subsequently visualized using a heatmap.

(I) Heatmap showed scaled chemokine expression levels across macrophage cell subtypes from ICC tumor and adjacent tissues.

(J) Heatmap showed functional enrichment scores across macrophage cell subtypes from ICC tumor, adjacent, and normal liver tissues.

Abbreviations: UMAP, Uniform Manifold Approximation and Projection; TF, transcription factor; ICC, intrahepatic cholangiocarcinoma; DEGs, differentially expressed genes.

**Supplementary Figure S5. TREM2^+^ macrophages are associated with adverse clinical outcomes and promote ICC progression.**
(A) TREM2 expression level across all cells and myeloid cells of ICC tumor and adjacent tissues by scRNA-seq analysis.

(B) Myeloid cell-related genes expression levels in macrophages from Yap-Akt-induced ICC by scRNA-seq.

(C) Survival curves (Kaplan-Meier) of TREM2^+^ macrophage-associated signatures in the EHBH-ICC-cohort (High group = 47, Low group = 44).

(D) IVIS optical imaging of tumor burden in *Trem2^-/-^* versus WT ICC mice induced by Yap-Akt-induced ICC mouse model.

(E) Line plot showed changes in TREM2^+^ macrophage marker expression during tumor progression.

(F) IVIS optical imaging of tumor burden in *F4/80*^Cre^-sh-*Trem2* versus control mice and the quantification of in vivo fluorescence signals obtained by live imaging (^***^*P* < 0.001, Mann-Whitney U-test).

**Supplementary Figure S6. Remodeling of the TME following *Trem2* knockout in ICC.**

(A) GO enrichment of genes that were upregulated in TREM2-high versus TREM2-low patients in EHBH-ICC-cohort.
(B) UMAP plots of all cells in scRNA-seq from normal liver, *Trem2^-/-^*, and WT ICC mice induced by Kras-P19.
(C) Marker genes expression levels detected by scRNA-seq analysis in all cell types (Alb was expressed in hepatocytes; Col1a1 in fibroblasts; Cd3e in T cells; Eng in endothelial cells; Cd19 in B cells; Krt19 in cholangiocytes; Ngp, Itgam, Ly6c2, and Siglech in myeloid cells).
(D) Swimming plot showing the cell type proportion detected in *Trem2^-/-^* versus WT tumors of mouse models (fold change > 0 indicates higher in *Trem2^-/-^* group).
(E) Marker gene expression in myeloid subclusters.
(F) Trem2^+^ macrophage proportions in Kras-P19-driven WT versus *Trem2^-/-^* tumors of mouse models (^****^*P* < 0.0001; Mann-Whitney U-test).
(G) Gating strategy for Trem2^+^ macrophages and myeloid cells in flow cytometry.
(H) *Trem2* expression detected by scRNA-seq analysis in myeloid cells of Kras-P19-induced ICC from the *Trem2^-/-^* and WT mouse models and normal liver.
(I) Gene expression levels detected by scRNA-seq analysis in kupffer cells.

(J-K) Dot plot of DEGs in Kupffer cells with the indicated clusters (J) and tissue types (K), identified by DEG analysis.

Abbreviations: ICC, intrahepatic cholangiocarcinoma; GO, Gene Ontology; UMAP, Uniform Manifold Approximation and Projection; WT, wild-type; DEG, differentially expressed gene.

**Supplementary Figure S7. TREM2^+^ macrophages promote tumor cell invasion in ICC via the PLAU-PLAUR axis.**
(A) Volcano plot of DEGs in malignant cells from *Trem2^-/-^* versus WT mice by DEG analysis.

(B) GO enrichment of genes that upregulated in WT malignant cells.

(C) Interaction strength between macrophage cell subtypes and malignant cells by CellphoneDB analysis.

(D) Ligand-receptor interactions between macrophage cell subtypes and malignant cells based on CellphoneDB analysis.

(E) UMAP plot showing PLAU and PLAUR expression levels in all cells.

(F) Violin plot showing PLAUR expression levels in tumor and adjacent epithelial cells.

(G) PLAU expression in macrophage cell subtypes was analyzed by scRNA-seq and visualized using violin plots.

(H) Immunohistochemical staining showed PLAUR expression levels in human ICC tumors and adjacent normal tissues.

(I) Co-localization of TREM2^+^ macrophages and PLAU in ICC tumor tissue showed by mIF staining.

(J) PLAUR co-expression with the malignant cell marker PanCK showed by mIF staining.

(K) Spatial transcriptomics was performed to identify PLAU and TREM2 co-enriched regions at the tumor boundary in tissues from two ICC patients.

(L) PLAU and PLAUR expression levels in ICC mouse tumors versus normal liver (^*^*P* < 0.05; ^****^*P* < 0.0001; ns, not significant; one-way ANOVA by Dunnett’s test).

(M) Kaplan-Meier survival curves of PLAU and PLAUR in the FuDan-ICC-cohort.

(N-O) Association of PLAUR (N) and PLAU (O) expression with clinicopathological features (FuDan-ICC-cohort).

Abbreviations: ICC, intrahepatic cholangiocarcinoma; DEG，differentially expressed gene; GO,Gene Ontology; WT, wild-type; mIF, multiplex immunofluorescence.

**Supplementary Figure S8. TREM2^+^ macrophages promote tumor cell invasion in ICC via the PLAU-PLAUR axis.**
(A) ELISA quantification of PLAU secretion by TREM2^+^ versus TREM2^-^ macrophages from human ICC tumor tissue (^****^*P* < 0.0001; Mann-Whitney U-test).

(B) Western blot analysis was performed to quantify PLAUR expression levels in human ICC cell lines.

(C) Transwell assay showing malignant cell invasion and migration after co-culture with TREM2^+^ macrophages with or without IPR-803 (^*^*P* < 0.05; ^**^*P* < 0.01; one-way ANOVA by Dunnett’s test).

(D) Transwell assay of malignant cell invasion/migration after treatment with PLAU recombinant protein and/or IPR-803 (^*^*P* < 0.05; ^**^*P* < 0.01; ^***^*P* < 0.001; ^****^*P* < 0.0001, one-way ANOVA by Dunnett’s test).

(E) Wound healing assay of malignant cells treated with PLAU recombinant protein and/or IPR-803 treatment (^*^*P* < 0.05; ^**^*P* < 0.01; one-way ANOVA by Dunnett’s test).

(F) Following AAV8-mediated targeted knockdown of Plaur expression in tumor cells, the silencing efficiency was validated by immunohistochemical scoring. (^***^*P* < 0.001; one-way ANOVA by Dunnett’s test).

(G) Morphological differences in liver tumors of ICC mice induced by Kras-p19 between the AAV8-*Ck19*^Cre^-sh-*PLAUR* group and the control group (^**^*P* < 0.01; one-way ANOVA by Dunnett’s test).

(H) Morphological differences in lung metastases of ICC mice between the AAV8-*Ck19*^Cre^-sh-*PLAUR* group and the control group (^*^*P* < 0.05; one-way ANOVA by Dunnett’s test).

(I) Kaplan-Meier survival curves of mice in the AAV8-*Ck19*^Cre^-sh-*PLAUR*-treatment group and the control group (Kras-p19-induced ICC).

(J-K) Morphology of tumors and lung metastasis in Kras-p19-induced ICC mice treated with IPR-803 or solvent (containing an equal volume of DMSO as the treatment group), and control mice (^*^*P* < 0.05; ^**^*P* < 0.01; ns, not significant; one-way ANOVA by Dunnett’s test).

Abbreviations: ICC, Intrahepatic cholangiocarcinoma; DMSO, dimethyl sulfoxide.

**Supplementary Figure S9. TREM2^+^ macrophages promote POSTN^+^ CAF formation at the tumor boundary to establish an immune barrier restricting CD8^+^ T cell infiltration in ICC.**
(A) UMAP plots of fibroblasts in *Trem2^-/-^* and WT tumors by Kras-p19-induced ICC and normal liver.

(B) Marker genes expression levels of fibroblast cell subtypes by scRNA-seq analysis.

(C) UMAP plot of fibroblasts in tumor and adjacent tissues. The myCAF subset is markedly absent in normal liver tissue. The red circle indicates the cell subpopulation of interest.

(D) Postn expression levels in different ICC mouse models and normal liver (^**^*P* < 0.01; ns, not significant; one-way ANOVA by Dunnett’s test).

(E) Spearman correlation of TREM2 with myCAF markers in the EHBH-ICC-cohort (Spearman rank correlation test).

(F-G) mIF staining analysis of CD8^+^ T cell spatial distribution at tumor boundaries with/without immune barriers (blue is nuclei staining; red is PanCK, indicating tumor epithelial cells; orange is POSTN, indicating POSTN-positive fibroblast-associated regions; yellow is TREM2, indicating TREM2-positive macrophage-related cells; white is CD8, indicating CD8-positive T cells; green is PD-1, indicating PD-1-positive immune cells).

(H-I) The pearson correlation score between TREM2 and POSTN fluorescence staining intensity in the existence (H) or non-existence (I) of an immune barrier.

(J) The expression levels of POSTN and FAP in human (left) and mice (right) ICC fibroblast subpopulations, indicating that POSTN and FAP have a high degree of expression similarity.

(K-L) Representative macroscopic images (K) of tumors and liver weights (L) in anti-Fap and control groups (Kras-p19-induced ICC; ^*^*P* < 0.05; Mann-Whitney U-test).

(M-O) Quantitative analysis of IHC scores for Postn and Cd8a, along with Sirius red staining scores, in ICC mouse tumor tissues following anti-Fap treatment (^****^*P* < 0.0001; Mann-Whitney U-test).

(P) CellPhoneDB analysis of ligand-receptor interactions between TREM2^+^ macrophages and fibroblast subtypes.

Abbreviations: ICC, intrahepatic cholangiocarcinoma; UMAP, Uniform Manifold Approximation and Projection; WT, wild-type; myCAF, myofibroblastic cancer-associated fibroblasts. mIF, multiplex immunofluorescence; ROI, region-of-interest; IHC, immunohistochemistry; SR, Sirius red staining.

**Supplementary Figure S10. TREM2^+^ macrophages promote immunosuppressive microenvironment in ICC by regulating CD8^+^ T cell exhaustion and Treg development.**
(A) Marker gene expression level in NK and T cell subsets by scRNA-seq analysis.

(B) Bubble plot of marker gene expression levels in NK and T cell subsets.

(C) UMAP plot showed NK and T cell subsets in *Trem2^-/-^* and WT Kras-P19-induced ICC mice.

(D) Proportion of NK and T cell subsets in *Trem2^-/-^* and WT TME with Kras-P19-induced ICC mice by cell proportion analysis.

(E) Spearman correlation of TREM2 with immunosuppressive markers in the FuDan-ICC-cohort (the mRNA expression levels were TPM-transformed and subsequently log2-normalized for correlation analysis).

(F) Flow cytometry analysis of Sell^+^Cd4^+^ T cell proportions in all Cd4^+^ cells (^***^*P* < 0.001; Mann-Whitney U-test).

(G) Flow cytometry analysis of Cd4^+^ and Cd8^+^ T cell subset ratios in Yap-Akt-induced ICC mice with *F4/80*^Cre^-sh-*Trem2* versus control group.

Abbreviations: ICC, intrahepatic cholangiocarcinoma; WT, wild-type; TME, tumor microenvironment.

**Supplementary Figure S11. Subset characteristics and spatial distribution of NK/T cells in the TME and peritumoral tissues of ICC.**

(A) UMAP plot of NK and T cells in the single-cell transcriptomic sequence cohort of ICC patients.

(B) Marker gene expression level in NK and T cell subsets of ICC tumor and adjacent tissues.

(C-D) UMAP plots of CD8^+^ T cell subsets of ICC tumor and adjacent and their marker expression levels.

(E-F) UMAP plots of CD4^+^ T cell subsets of ICC tumor and adjacent and their marker expression levels.

(G-H) UMAP plots of NK cell subsets of ICC tumor and adjacent and their marker gene expression levels.

(I) Distribution differences of NK and T subsets in tumor versus peritumoral tissues (^*^*P* < 0.05; ^**^*P* < 0.01; ^***^*P* < 0.001; Mann-Whitney U-test).

Abbreviations: ICC, intrahepatic cholangiocarcinoma; UMAP, Uniform Manifold Approximation and Projection.

**Supplementary Figure S12. Characteristics of Treg cells in the ICC TME.**

(A) Heatmap of the interaction strength between CD4^+^ T cells and macrophages in tumor tissue by CellphoneDB and showing by heatmap.

(B) Bubble plot of ligand-receptor pairs between macrophage subtypes and Treg subsets by CellphoneDB analysis.

(C) DEGs between TNFSF14^hi^ Treg and TIGIT^hi^ Treg cells by DEG analysis.

(D) UMAP plots of three T cell subsets and the expression level of CCR8, TNFRSF4, and TNFRSF9 by scRNA-seq analysis.

(E) Distribution of TIGIT^hi^ Treg and TNFSF4^hi^ Treg cells in the GSE211068 dataset.

(F) Pseudotime dynamics of TNFRSF8, REL, BATF, and Treg functional markers (Monocle2).

(G) Pseudotime trajectory of naive CD4^+^ T cell differentiation into Treg subsets.

(H) Gating strategy for naive CD4^+^ T and nTreg cells by flow cytometry.

(I) Ligand-receptor interactions between TREM2^+^ macrophages and naive CD4^+^ T/TNFSF14^hi^ Treg/TIGIT^hi^ Treg cells.

(J) Spearman correlation between TNFSF13B and CCR8 expression in the Xena pan-cancer cohort (Spearman rank correlation test).

Abbreviations: ICC, intrahepatic cholangiocarcinoma; DEG, differentially expressed gene; UMAP, Uniform Manifold Approximation and Projection.

**Supplementary Figure S13. TREM2^+^ macrophages promote immunosuppressive microenvironment in ICC by regulating CD8^+^ T cell exhaustion.**

(A) After CellPhoneDB analysis, a chord diagram was used to illustrate the interaction strength between macrophage subsets and exhausted CD8^+^ T cells in tumor tissues. Thicker connecting lines indicate stronger interactions.

(B-C) Heatmap (B) and chord diagram (C) showed interaction strength between macrophage subtypes and CD8^+^ Tex cells by CellphoneDB.

(D) Bubble plot of ligand-receptor pairs between CD8^+^ Tex and macrophage subtypes by CellphoneDB.

(E) Immune cell correlations in adjacent tissues come from scRNA-seq data cohort (Spearman correlation).

(F) Dot plot of genes correlated with CD8^+^ Tex in adjacent tissues.

(G) PCA plot of bulk RNA-seq data from CD8^+^ T cells that co-cultured with macrophage sub-cell types.

(H) GO enrichment results of genes upregulated in CD8^+^ T cells after TIMD4^+^ macrophage co-culture.

Abbreviations: ICC, intrahepatic cholangiocarcinoma; PCA, principal component analysis; GO, Gene Ontology.

**Supplementary Figure S14. Targeting Trem2 and Timd4 to enhance anti-Pd-1 efficacy in ICC.**

(A) PCA plot of bulk RNA-seq from anti-Csf1r-antibody-treated versus control group.

(B) Survival curves (Kaplan-Meier) of CSF1R in the FuDan-ICC-cohort.

(C) IVIS optical imaging and quantification of tumor burden in Yap-Akt-induced ICC mice across indicated treatment groups (one-way ANOVA by Dunnett’s test).

(D-G) Flow cytometric analysis of differences in immune cells (Cd8 denotes CD8⁺ T cells [D]; Pd-1 denotes exhausted T cells [E]; Foxp3 denotes Tregs [F]; Ly6C denotes myeloid cells [G]) among indicated treatment groups.

(H) Ly6c^+^ myeloid cell proportions across indicated treatment groups (^**^*P* < 0.01; ^****^*P* < 0.0001; ns, not significant; one-way ANOVA by Dunnett’s test).

(I) Survival curves (Kaplan-Meier) of Yap-Akt-induced ICC mice under therapeutic interventions.

Abbreviations: ICC, intrahepatic cholangiocarcinoma; PCA, principal component analysis; Treg, regulatory T cell.

**Supplementary Figure S15. In vivo safety evaluation of the drug.**

(A) Peripheral blood counts and hepatic/renal function profiles in mice (after five administrations of different drugs in healthy mice, blood was collected via the retro-orbital venous plexus. The blood samples were gently mixed and divided into two aliquots-one for complete blood count analysis and the other for serum separation to assess hepatic and renal function; ^*^*P* < 0.05; ^**^*P* < 0.01; ^***^*P* < 0.001; ns, not significant; one-way ANOVA by Dunnett’s test).

(B) H&E-stained pathological sections of major organs (heart, liver, spleen, lungs, and kidneys) from mice treated with different drug regimens (following drug administration, H&E staining was performed on the heart, liver, spleen, lungs, and kidneys of the mice to evaluate potential pathological alterations).

Abbreviations: H&E, Hematoxylin and eosin.
